# Antineoplastic Activity of a Novel Trispecific Single-Chain Antibody Targeting the hERG1/β1 Integrin Complex and TRAIL Receptors

**DOI:** 10.1158/1535-7163.MCT-24-0646

**Published:** 2025-06-18

**Authors:** Claudia Duranti, Jessica Iorio, Chiara Capitani, Tiziano Lottini, Michele Martinelli, Julia Roosz, Nicole Anderle, Tengku Ibrahim Maulana, Peter M. Loskill, Rossella Colasurdo, Cesare Sala, Lara Magni, Annarosa Arcangeli

**Affiliations:** 1 Department of Experimental and Clinical Medicine, University of Florence, Florence, Italy.; 2 MultiFacility 3R, Department of Experimental and Clinical Medicine, University of Florence, Florence, Italy.; 3 MCK Therapeutics Srl, Pistoia, Italy.; 4 Department of Medical Biotechnologies, University of Siena, Siena, Italy.; 5 NMI Natural and Medical Sciences Institute at the University of Tübingen, Reutlingen, Germany.; 6 TestingDepartment for Microphysiological Systems, Institute of Biomedical Engineering, Eberhard Karls University Tübingen, Tübingen, Germany.

## Abstract

Targeted therapies and immunotherapies have largely improved cancer treatment in the last years. One of the most promising approaches is the induction of tumor apoptosis by TRAIL through its binding to apoptosis-inducing receptors DR4 and DR5 on the plasma membrane of target cells. However, some constraints (e.g., the short *in vivo* half-life and the poor activity on DR5 receptors) hinder the use of naked, soluble forms of TRAIL. Previous studies have shown that fusing TRAIL sequences with antibody-based moieties may represent a novel and efficacious strategy to overcome such hindrances. On these bases, novel TRAIL-related anticancer therapeutic strategies are being developed. In the present article, we describe a novel antibody represented by a single-chain diabody directed against a cancer-specific target, i.e., the hERG1/β1 integrin complex—scDb-hERG1-β1—fused with three TRAIL sequences. The scDb-hERG1-β1-TRAIL antibody combines the specific targeting and downregulation of cancer-specific signaling pathways by scDb-hERG1-β1 with the proapoptotic activity triggered by TRAIL. We provide substantial evidence of the efficacy of the scDb-hERG1-β1-TRAIL antibody to decrease tumor growth triggering apoptotic cell death *in vitro* in breast cancer cells as well as *in vivo* in a mouse model of triple-negative breast cancer. Being characterized by a favorable pharmacokinetic and toxicity profile, the scDb-hERG1-β1-TRAIL antibody can be proposed for the treatment of difficult-to-treat cancers, such as triple-negative breast cancer, which express the hERG1/β1 complex and TRAIL receptors.

## Introduction

Over the past 20 years, targeted therapies and immunotherapies have largely improved cancer treatment (1, 2) with the use of therapeutic monospecific or bispecific antibodies (3, 4). Immunotherapies have been further improved by the development of antibody fragments, characterized by a greater permeability within tumor masses (5). In this scenario, the targeting of the apoptotic machinery has attracted many efforts, with TNF first and then TRAIL (6). TRAIL is a type II transmembrane protein expressed on different immune and tumor cells which is deeply involved in antitumor activity (7). TRAIL is capable of selectively activating the extrinsic apoptotic pathway, once bound to “apoptosis-inducing” receptors TRAIL-R1, DR4 and TRAIL-R2, DR5 (8). Because of these activities, TRAIL is a potential candidate for cancer therapy, through either recombinant soluble TRAIL proteins, such as the trimeric TRAIL (9, 10), or TRAIL-R agonists (11). Soluble TRAILs showed little to no toxicity when administered systemically to mice and induced apoptotic cell death in malignant cells (12). However, their short half-life in serum [in the range of minutes (13)] and unwanted actions on normal cells or the occurrence of resistance (14) have hampered their therapeutic use so far. Furthermore, products endowed with agonistic activity or TRAIL-R DR4 and DR5 (15) have shown very low efficacy once administered *in vivo* (16). Overall, an improvement of the pharmacokinetic properties of TRAIL proteins, such as their combination with specific tumor-targeting moieties, is envisaged to increase the therapeutic potential of TRAIL-based therapies. Indeed, novel products have been developed combining TRAIL, particularly its trimeric form, with either full-length or single-chain variable fragment (scFv) antibodies. All these products show a more specific tumor killing and enhanced specific bioactivity compared with soluble TRAIL proteins. Examples of such products are fusion proteins of TRAIL with a scFv targeting ErbB2 (17), or a scFv specific for the human multidrug resistance protein 3, scFvM58-sTRAIL (18), or Meso–TR3, a fusion protein between native mesothelin and TR3, a genetically fused trimer of TRAIL (19).

In this scenario, we have generated a TRAIL-based immunoconjugate in which the targeting moiety is represented by a single-chain diabody (scDb-hERG1-β1) directed against two proteins, hERG1 and the β1 integrin, which form a complex in cancer cells (20), fused with three TRAIL sequences which bind to TRAIL receptors (TRAIL-R), hence generating a “trispecific” antibody: scDb-hERG1-β1-TRAIL. The scDb-hERG1-β1 targets with high affinity the hERG1/β1 integrin complex which is selectively expressed in cancer cells (20). The diabody has an antineoplastic activity per se, being capable of decreasing cell proliferation and migration/invasiveness (bioRxiv 2024.07.02.601509; ref. 20) through the inhibition of the PI3K/Akt pathway downstream to the hERG1/β1 integrin complex (Duranti and colleagues, unpublished observations December 2024; refs. 21, 22). The scDb-hERG1-β1-TRAIL combines these activities with the proapoptotic activity triggered by TRAIL. We provide evidence that the scDb-hERG1-β1-TRAIL antibody can induce apoptotic cell death both *in vitro* and *in vivo* in breast cancer cells, sparing normal cells, with particular efficacy in triple-negative breast cancer (TNBC) cells which express the hERG1/β1 integrin complex.

## Materials and Methods

### Reagents

For PCR, KOD Hot Start DNA Polymerase (Novagen, cat. 71086-5) was used. pPIC9K (Thermo Fisher Scientific, cat. V17520): expression vector for the scDb-hERG1-β1 antibody in *Pichia pastoris*. pEGFP-TRAIL (plasmid #10953, Addgene; RRID: Addgene_10953): plasmid from which TRAIL sequence was isolated via PCR. pSEcTag2A (Thermo Fisher Scientific, cat. V90020): expression vector for the scDb-hERG1-β1-TRAIL antibody in CHO-K1 cell line. Cell lysis buffer for protein extraction was from Cell Signaling Technology. Composition of lysis buffer: NP40 (150 mmol/L), NaCl (150 mmol/L), Tris-HCl pH 8 (50 mmol/L), EDTA pH 8 (5 mmol/L), NaF (10 mmol/L), Na4P2O7 (10 mmol/L), Na3VO4 (0.4 mmol/L), and a protease inhibitor cocktail (cOmplete Mini, Roche). The following antibodies were used: rabbit polyclonal Bax antibody (N-20, Santa Cruz Biotechnology, cat. sc-493; RRID: AB_2227995) at a final dilution of 1:200 for Western blot (WB); mouse cleaved caspase-8 mAb (H-134, Santa Cruz Biotechnology, cat. sc-7890; RRID: AB_2068330) at a final dilution of 1:500 for WB; rabbit polyclonal NF-kB antibody (Santa Cruz Biotechnology, sc-109; RRID: AB_632039) at a final dilution at 1:200 for immunofluorescence; mouse mAb AKT1/2/3 (H-136; Santa Cruz Biotechnology, cat. sc-8312; RRID: AB_671714) at a final dilution 1:500 for WB; mouse mAb p-Akt1/2/3 (Thr 308; Santa Cruz Biotechnology, cat. sc-271966; RRID: AB_10715102) at a final dilution 1:500 for WB; anti–cyclin E (Santa Cruz Biotechnology, cat. sc-247; RRID: AB_627357) at 1:500 for WB; anti–cyclin D (Cell Signaling Technology, cat. 2978; RRID: AB_2259616) at 1:1,000 for WB; mouse tubulin mAb antibody (Sigma-Aldrich, cat. T9026; RRID: AB_477593) at 1:500 dilution for WB; rabbit polyclonal anti-DR4 antibody (Sigma-Aldrich, cat. AB16955; RRID: AB_11215620) at 1:500 dilution for WB; rabbit polyclonal anti-DR5 antibody (Sigma-Aldrich, cat. AB16942; RRID: AB_11213162) at 1:400 dilution for WB; bispecific antibody directed against the hERG1/β1 complex, scDb-hERG1-β1 (MCK Therapeutics) at 0.9 µmol/L for viability, apoptosis, proliferation, and calcein/propidium iodide (PI) and lactate dehydrogenase (LDH) assays; and mouse mAb for albumin (F-10, Santa Cruz Biotechnology, cat. sc-271605; RRID: AB_10647230) at 1:500 dilution for WB. An anti-TRAIL antibody (Thermo Fisher Scientific, cat. PA1-955; RRID: AB_2256246) was used for ELISA at a final dilution of 1:100. An anti-6xHis antibody and the mouse IgG–horseradish peroxidase (HRP) conjugate (Sigma-Aldrich, cat. A4416; RRID: AB_258167) were used for ELISA at 1:500. For annexin/V assay, BV421 annexin V (BD Biosciences, cat. 563973; RRID: AB_2869538) and 5 µL of 7-ADD (BD Biosciences, cat. 559925; RRID: AB_2869266) have been used. The following secondary antibodies were used: IRDYe 800 CW anti-mouse and anti-rabbit secondary antibodies (LI-COR Biosciences, cat. 926-32210; RRID: AB_3096013, 926-32211; RRID: AB_6218431) at 1:20.000 for WB and anti-mouse IgG–HRP conjugate (Sigma-Aldrich, cat. A4416; RRID: AB_258167) at 1:500 for ELISA. Aspartate transaminase (AST) and alanine aminotransferase (ALT) activity assays were used to monitor protein levels (Thermo Fisher Scientific, cat. MAK055 and cat. MAK052). The anti-6xHis-tag antibody (4D11, Abcam, cat. ab5000; RRID: AB_304722) was used at 1:250 dilution for IHC. A terminal deoxynucleotidyl transferase–mediated dUTP nick end labeling (TUNEL) assay kit was used (Abcam, HRP-DAB, cat. AB206386).

### Solutions

Elution buffer: 20 mmol/L sodium phosphate, 500 mmol/L NaCl, and 500 mmol/L imidazole, pH 7.3.

### Development of the scDb-hERG1-β1-TRAIL antibody

**Figure 1.**
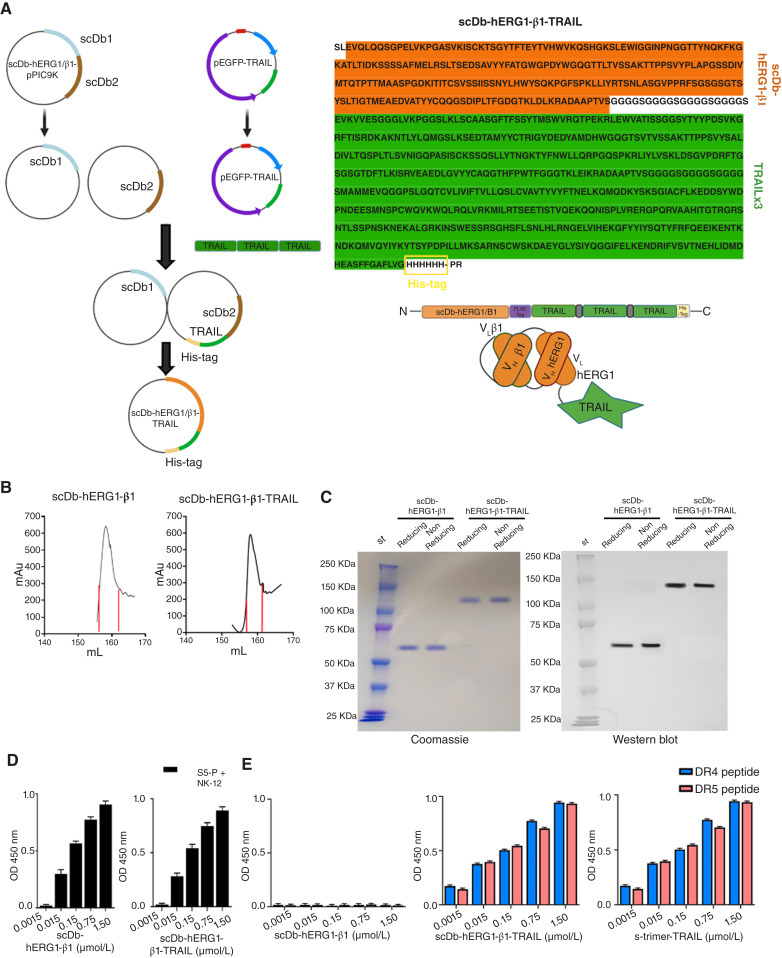
Biochemical characterization of the scDb-hERG1-β1-TRAIL antibody. **A,** Flow chart of the procedure for developing the scDb-hERG1-β1-TRAIL (left). Amino acid sequence and schematic representation of the scDb-hERG1-β1-TRAIL (right). **B,** Representative chromatograms of scDb-hERG1-β1 and scDb-hERG1-β1-TRAIL. **C,** Representative Coomassie brilliant blue staining (left) and Western blot (right) under reducing and nonreducing conditions of scDb-hERG1-β1 and scDb-hERG1-β1-TRAIL. **D,** Peptide ELISA using a 1:1 mix of hERG1 S5-P peptide (hERG1) and NK-12 peptide (β1 integrin) with scDb-hERG1-β1 and scDb-hERG1-β1-TRAIL. **E,** Peptide ELISA using the DR4 (light blue) and DR5 (pink) peptides with scDb-hERG1-β1, scDb-hERG1-β1-TRAIL, and s-trimer-TRAIL. Values are expressed as OD[224711]  450 nm and are means ± SEM of three independent experiments. mAu, milli-absorbance unit. OD, optical density. [**A,** Created in BioRender. Duranti, C. (2025) https://BioRender.com/bqymq1f.]

To develop the scDb-hERG1-β1-TRAIL antibody, the following steps were followed: (i) the sequence encoding for scDb-hERG1-β1 was isolated from the pPIC9K vector (20) via two PCR steps, obtaining “scDb1” and “scDb2” sequences, (ii) three TRAIL sequences were isolated from the pEGFP-TRAIL vector (Addgene, cat. 10953; RRID: Addgene_10953) via three sequential PCRs, and (iii) scDb2 was first linked to TRAIL sequences and then scDb1 was linked to scDb2-TRAIL by two PCRs. The two external primers, scDb-FOR and TRAIL-REV, carried HindIII and NotI restriction sites, respectively, which were used to insert the construct into the mammalian cell expression vector pSEcTag2A (Thermo Fisher Scientific, cat. V90020), hence obtaining the final pSEcTag2A-scDb-hERG1-β1-TRAIL construct. The pSEcTag2A-scDb-hERG1-β1-TRAIL antibody was first used to transform DH5α *Escherichia coli* cells and verified by sequencing and then transfected (4 mg per 10^5^ cells) into CHO-K1 cells (ATCC, cat. CCL-61; RRID: CVCL_0214) using Lipofectamine 2000 (Thermo Fisher Scientific, cat. 12566014). Twenty-four hours after transfection, cells were split 1:3, and then the Zeocin (Thermo Fisher Scientific, cat. R25001) selective agent was added at 30 μg/mL. Supernatants (usually 3 L) from CHO-K1-pSEcTag2A-scDb-hERG1-β1-TRAIL cells were purified by affinity chromatography using an ÄKTA start protein purification system (Cytiva, cat. 29022094) with HisTrap HP 1 mL columns (Cytiva, cat. GE29-0485-86) at a running speed of 1 mL/minute. Elution was performed using a linear imidazole gradient of elution buffer. Analysis was accomplished using UNICORN v7.0 software. The fractions in which the protein was detected were collected, pooled, and dialyzed against PBS using a Slide-A-Lyzer dialysis cassette (Thermo Fisher Scientific, cat. 66830). The protein was first quantified by NanoDrop (Thermo Fisher Scientific, cat. ND-ONE-W), then concentrated through an Amicon Ultra system (Merck Millipore, cat. C134281), and then gel-filtered using Superdex 75 HR 10/30 (GE HealthCare Life Sciences, cat. 17116601). The flow chart of the procedure is in Fig. 1A, and additional details can be found in the “Materials and Methods” section and in Supplementary Fig. S1.

### Labeling of scDb-hERG1/β1 with indocyanine green

Amine-reactive indocyanine green (ICG)-NHS (ICG-NHS, Iris Biotech) dye equipped with succinimidyl (NHS) ester reacts efficiently with primary amines of proteins to form stable dye–protein conjugates. ICG-NHS was first dissolved in anhydrous DMSO (Sigma-Aldrich). The scDb-hERG1-β1 solutions in PBS were incubated with ICG at molar ratios of ICG:scDb of 5 (condition 1), 10 (condition 2), and 20 (condition 3; 5×, 10×, and 20×) in conjugation buffer (0.002 MNaHCO_3_ + 0.048 MNa_2_CO_3_ + 0.15 MNaCl, pH 8.5) for 1 hour in the dark at 37°C with gentle mixing. Purification was performed using PD-10 desalting columns (Cytiva) according to the manufacturer’s instructions.

The concentration of scDb-hERG1-β1 and scDb-hERG1-β1-TRAIL was calculated for all the three above-mentioned conjugation conditions using the following equation: protein concentration = A protein × protein molecular weight/∑ protein, in which A protein = A280 − Amax × 0.07, with Amax (absorbance of the dye at ≤ max) determined at 800 nm and 0.07 = contribution of the dye to the absorbance at A280 (CF ICG-NHS = 0.07). Subsequently, the degree of labeling (D.O.L., corresponding to the number of dye molecules present on the labeled protein) was calculated for the three conditions using the following equation: D.O.L. = (Amax × protein molecular weight)/([protein] × ∑dye), in which ∑dye = 223,000 cm^−1^ (mol/L)^−1^. The D.O.L. for each condition turned out to be 2.01 (condition 1), 3.22 (condition 2), and 4 (condition 3); thus, the preparation with the highest D.O.L. was used for *in vivo* experiments.

### Cell cultures

Cells were obtained from the ATCC in 2014. Cell authentication has been performed through morphology test by microscopy, growth curve analysis, and *Mycoplasma* analysis. All cells derive from female donors. MCF-10A (RRID: CVCL_0598), MCF-7 (RRID: CVCL_0031), MDA-MB-231 (RRID: CVCL_0062), and HEK293 (RRID: CVCL_0045) cell lines were cultured in DMEM (Euroclone, cat. ECM0101L), with 4 mmol/L L-glutamine (Euroclone, cat. ECB3000) and 10% (5% for MDA-MB-231 cells) FBS (European Union–approved Euroclone, cat. ECS5000L). U2932 (RRID: CVCL_1896) cells were cultured in RPMI (Euroclone, cat. ECB9006L), 2 mmol/L L-glutamine, and 10% FBS. HEK293 cells expressing the hERG1 construct (HEK–hERG1; ref. 23) were maintained in DMEM, 4 mmol/L L-glutamine, and 10% FBS supplemented with 0.8 mg/mL of geneticin (G418; Thermo Fisher Scientific, cat. 10131035). CHO-K1-scDb-hERG1/β1-TRAIL cells were cultured in HAM’s F-12 (Euroclone, cat. ECM0090L), 2 mmol/L of L-glutamine, and 10% FBS, supplemented with 30 µg/mL Zeocin (Thermo Fisher Scientific, cat. R25001). All the cells were cultured at 37°C with 5% CO_2_ in a humidified atmosphere. We certify that all cell lines used in the present study were routinely screened, once a month and before performing *in vivo* experiments, for *Mycoplasma* contamination, through PCR, and only *Mycoplasma*-negative cells were used. Cells were passed to the utmost 10 times between thawing and freezing.

### Cell viability assay

Cell viability was measured by the trypan blue exclusion test as in (24). Cells were counted after trypan blue (Merck Millipore, cat. T8154) addition using LUNA-II Automated Cell Counter (Logos, cat. LUC-04-00823).

### Apoptosis assay

Apoptosis was determined using the annexin-V/PI test (Annexin-V FLUOS Staining Kit, Roche Diagnostics, cat. 11 858 777 001) as previously described in (24). The following stages were determined in the dot plots: live (Q1), early apoptotic (Q2), late apoptotic (Q3), and necrotic (Q4).

### Peptide- and cell-based ELISAs

Peptide-ELISA was performed following the procedure described in (20), coating the wells with the different peptides listed in Supplementary Table S3. Cell-based ELISA was performed as described in (20) on live cells. In either tests, sdDb-hERG1-β1, scDb-hERG1-β1-TRAIL, and the soluble trimer (s-trimer-)TRAIL were added at the concentrations listed in parentheses: scDb-hERG1-β1 (0.1, 1,10, 50, and 100 mg/mL), scDb-hERG1-β1-TRAIL (0.2, 2, 20, 100, and 200 mg/mL), and s-trimer-TRAIL (0.1, 1,10, 50, and 100 mg/mL). Following incubation (either 2 or 8 hours for the cell-based ELISA only) the anti-6xHis–HRP antibody (Merck, cat. A7058; RRID: AB_258326 1:250) was added, and the colorimetric reaction was evaluated at 450 nm.

### Competitive ELISA

Competitive cell ELISAs were performed on MDA-MB-231 cells. First test: MDA-MB-231 cells were seeded in the absence or presence of s-trimer-TRAIL (1 µmol/L) for 1 hour, then incubated with either scDb-hERG1-β1 (1.5 µmol/L) or scDb-hERG1-β1-TRAIL (1.5 µmol/L) for 1 hour, and then revealed by anti-His antibody. Second test: MDA-MB-231 cells were seeded in the presence of s-trimer TRAIL (1 µmol/L) for 1 hour, then incubated with scDb-hERG1-β1-TRAIL or nothing, and then revealed with the anti-His or anti-TRAIL antibody (final dilution 1:100, see the “Reagents” section) and then with mouse IgG–HRP conjugate as a secondary antibody (see the “Reagents” section).

### Cell cultures in microfluidic chips

#### Tumor-on-chip concept, design, and fabrication

The study was conducted using a previously established tumor-on-chip system (2). Briefly, the chip consists of two stacked microfluidic channels separated by an isoporous, semipermeable polyethylene terephthalate (PET) membrane (5 μm pore size: rP = 5 μm; ρP = 6 × 10^5^ pores per cm^2^; TRAKETCH PET 5.0 p S210 × 300, SABEU GmbH & Co. KG), which was functionalized by a plasma-enhanced, chemical vapor deposition process.

The tumor chambers (1 mm in diameter, 0.3 mm in height, and six chambers per chip) branch off a main injection channel (0.2 mm in height) at a 45 degrees angle and a high-resistance channel toward the outlet port, which forces the aggregates/organoids to sequentially enter the tumor chambers during the injection process. This concept and design enable the integration of any aggregate/organoid below 0.2 mm in size. The medium channel is situated right above the tumor chambers and is separated by a porous PET membrane (Supplementary Fig. S4B).

The 200- to 300-μm-high tumor chambers and medium channels were microstructured via replica molding of polydimethylsiloxane (PDMS; Sylgard, cat. 184, Dow Corning) on two differently patterned master wafers fabricated via photolithographic processes. For the replica molding, PDMS was homogeneously mixed in a 10:1 (elastomer base: curing agent) mass ratio and then degassed in a desiccator to remove air bubbles. Two different replica molding approaches were conducted: (i) Standard molding comprising pouring of PDMS prepolymer solution onto the silicon wafer master mold was used to obtain 3-mm-thick PDMS pieces featuring the medium channel structures. PDMS was cured at 60°C for 4 hours. After curing, the PDMS modules were cut to the size of the chip, and ports were pierced using a biopsy punch (Disposable Biopsy Punch, 0.75 mm diameter; cat. 504 529; World Precision Instruments) to access the channels (for both cell injection and medium perfusion). (ii) Exclusion molding was used to fabricate the bottom layer featuring through-hole structures, the tumor chambers. In this study, PDMS prepolymer solution was poured onto the silicon wafer master mold, which was then clamped against a 5-mm-thick PMMA disk to produce a 0.3 mm thin layer with hole structures. PDMS was cured at 60°C for 2 hours. Once the PDMS parts were cured, they were cleaned using isopropanol followed by deionized water and blow-dried with an air gun. Finally, the microfluidic modules were assembled in three consecutive bonding steps: (i) tumor layer to glass coverslip, (ii) medium layer to the membrane, and (iii) tumor layer to medium layer. For all steps, bonding was achieved by oxygen plasma activation (75 W, 0.2 cm^3^ m^−1^ O_2_; Diener Zepto, Diener electronic GmbH + Co. KG) for 24 seconds. Bonded parts were baked at 60°C for at least 30 minutes after each bonding step and overnight after the entire chip was assembled. All chips were O_2_–plasma-treated (75 W, 0.2 cm^3^ m^−1^ O_2_) for 5 minutes to sterilize and hydrophilize the PDMS surface before cell injection.

1. Tumor aggregate generation and integration into the tumor-on-chip system

Cells (either MDA-MB-231 or PANC-1) were detached, counted, and subsequently resuspended in a 1:1 mixture of BME (Basement Membrane Extract2 Cultrex Reduced Growth Factor, Bio-Techne, cat. 3532-010-02) and cell culture medium on ice. After carefully removing the medium from the agarose mold, 200 µL of cell–gel suspension was added to each well. Following a centrifugation step at 900 × *g* for 10 minutes at 4°C, the plate was incubated for 30 minutes in a humidified 37°C incubator for complete gelation. At the end, 1 mL of warm culture medium was added into each well. After 3 days of culture, ∼100-µm-sized aggregates were generated. Chips were then primed by first flushing three times with 70% ethanol and then three times with sterile PBS. Chips were stored at room temperature (RT) in the sterile bench until cell seeding. The cell aggregates were retrieved from the well after dissociating the gel with 500 µL of cold dispase (2 mg/mL; Merck, cat. 04942078001). Aggregates were centrifuged to create a pellet, which was then resuspended in a 3-D Life SG-Dextran hydrogel solution (10× CB, SG Dextran, cat. G93-1, RGD Peptide, cat. 09-P-003 and CD-Link, cat. L60-3; Cellendes GmbH). Afterward, 10 µL of the aggregate–gel solution was loaded into the chip through the tissue inlet. Finally, the tissue inlet and outlet were plugged with a 0.7 mm precut wire (Menzanium, cat. 8466), and the chip was incubated at 37°C for at least 30 minutes to let the gel solidify. Spheroids from one AggreWell were typically used to load 3 to 4 chips.

2. Tumor-on-chip perfusion and withdraw treatment

Before starting the cell culture, the medium was warmed up, degassed, and filter-sterilized by applying negative pressure on a 50-mL Falcon tube via a Steriflip conical filter unit (Merck, cat. SCGP00525) for 30 minutes while simultaneously warming up the medium in a 37°C water bath. After that, the medium temperature and gas saturation were equilibrated by placing the Falcon tube in the humidified incubator with a loosened cap for 30 minutes. Afterward, 2.5 mL of medium was loaded into a syringe, followed by connecting the syringe to the inlet tubing. Syringes were then mounted onto the pump. Each chip was perfused with medium from a syringe. Prior to connecting the inlet tubing into the chip’s inlet, the tubing was primed with medium using the pump. Chips were connected to the pumps and downstream 5-mL Eppendorf tubes as a reservoir to collect the effluent. The entire setup was then transferred into the incubator, and the pump flow rate was set to 20 μL/hours.

After preparing tubings (previously autoclaved), syringes, and syringe pump (12 channel; Landgraf Laborsysteme, cat. 106720190) in the sterile bench, the syringes were mounted on the syringe pump on a fully dispensed plunger position. The syringe diameter was set on the pump. The inlet tubings were carefully connected to the media channel inlet. Using the P100 pipette tip, 20 μL of medium were administered on the inlet. The flow rate was set to 100 μL/hours and the pump in “withdraw” mode to generate negative pressure. Once the liquid level on the pipette tip decreases normally, 400 μL of medium were added to the pipette tip. At this point, the pump flow rate was set to 20 μL/hours and the whole setup was moved into the incubator.

3. Calcein/PI assay

3D cell cultures of PANC-1, MDA-MB-231, and HCT-116 were treated with the IC_50_ concentration of antibody scDb-hERG1-β1-TRAIL through a linear withdrawing system for 24 hours. A mix containing calcein (Thermo Fisher Scientific, cat. C1430) in a dilution of 1:400 and PBS was prepared. After placing an empty 100-μL tip in the media channel outlet, 100 μL of the staining solution were added to the chip and incubated for 40 minutes at RT. After this, the chip was washed with 100 μL of PBS. Finally, a 100 μL of solution mix of PI) (Thermo Fisher Scientific, cat. P4170-10MG) in a dilution of 1 mg/mL and PBS were added and incubated for 5 minutes at RT. Live imaging was then performed using a confocal microscope (LSM 880, Carl Zeiss).

4. LDH assay

3D cell cultures of PANC-1, MDA-MB-231, and HCT-116 were treated with the IC_50_ concentration of antibody scDb-hERG1-β1-TRAIL through a linear pushing perfusion system. Samples of the culture medium were collected form the reservoirs at the desired experimental time points (24, 48, and 96 hours) and by removing 2 to 5 μL of medium and diluting into 48 to 95 μL LDH storage buffer (Promega, cat. J2381). Maximum LDH release control, required to calculate the cytotoxicity level, is obtained by adding 2 μL of 10% Triton X-100 (per 100 μL original volume) to the vehicle-only wells, mixing, and incubating for at least 10 to 15 minutes before sample removal.

After collecting and diluting all samples, 50 μL of diluted sample were transferred into a 96-well opaque-walled, nontransparent assay plate (with clear or opaque bottom). Fifty μL of LDH detection reagent (Promega, cat. J2381) were added to each well. Plates were then incubated for 60 minutes at RT. Luminescence was recorded after 30 to 60 minutes after adding LDH detection reagent.

Cytotoxicity (%) = 100 × (Experimental LDH release - Medium background)/(Maximum LDH release control - Medium background)

#### Protein extraction, Coomassie, WB, and coimmunoprecipitation

Protein extraction and quantification were performed essentially as reported in (23). The NP-40 based lysis buffer (see reagents in Supplementary Materials and Methods) contained the protease inhibitor cocktail (Roche, cat., 04693124001). After boiling the extracted proteins for 5 minutes at 95°C, SDS-PAGE was performed for 1 hour at 100 V in Tris–glycine–SDS running buffer (Bio-Rad, cat. 1610772). For Coomassie, each polyacrylamide gel (gradient 4%–20%) was stained with Coomassie Brilliant Blue (Bio-Rad, cat. 1610400). For WB, (25), proteins were electrophoresed on a 7.5% polyacrylamide gel, transferred onto an activated polyvinylidene difluoride membrane, and then incubated with the primary and secondary antibodies listed in Supplementary Materials and Methods. For coimmunoprecipitation, 1.5 mg of extracted proteins were precleared with Protein A/G Plus-Agarose beads (Santa Cruz, cat. sc. 2003) and then immunoprecipitated with the TS2/16 antibody (Campoverde, cat. 303010; ref. 25).

#### Densitometric analysis

Proteins were quantified by optical densitometry using ImageJ and plotting the data with OriginPro8 software (OriginLab Corporation). For the quantification of the hERG1/β1 integrin complex, the signal of the coimmunoprecipitated hERG1 protein was divided by the signal of the protein used for immunoprecipitation (β1 integrin) and then normalized to the signal of the corresponding protein in the total lysate (β1 integrin input).

#### Animal studies

1. Pharmacokinetics

Eight-week-old immunocompetent FVB female mice (Envigo RMS S.r.l, Italy) were injected with 160 µg (8 mg/kg) of scDb-hERG1-β1-TRAIL antibody, and blood samples were collected from the tail vein at 15′, 30′, 60′, 180′, 6, 24, and 48 hours after antibody injection. Each sample was spun at 12,000 rpm for 5 minutes, and the resulting plasma was stored at −80°C until analyzed. The plasma concentration of scDb-hERG1-β1-TRAIL antibody was determined by WB (see “Materials and Methods”), using the anti-TRAIL antibody (Thermo Fisher Scientific, cat. PA1-955; RRID: AB_2256246) 1 µg/mL in PBS-5% BSA to reveal the scDb-hERG1-β1-TRAIL antibody, followed by anti-rabbit IgG (LI-COR Biosciences, cat. 926-32211 Lincoln, NE; RRID: AB_6218431) 1:20.000 in PBS-5% BSA. The serum stability against proteolytic activities of the scDb-hERG1-β1-TRAIL antibody was assessed using 150 mg of the antibody. The scDb-hERG1-β1-TRAIL antibody was incubated at 37°C in mouse serum up to 96 hours (0, 15′, 30′, 2, 12, 2, 48, 72, and 96 hours), and its concentration was determined through a WB and densitometric analysis.

2. Half-life determination

The half-lives for the elimination phase were determined using Origin 7.0 Software by fitting the last four data points into the first-order equation, *T*1/2 = (Δ*t*/*t*1 − *t*0)/Δ*C*, in which (Δ*t*/*t*1 − *t*0) represents the slope of the curve and Δ*C* represents the value corresponding to the half of the antibody concentration at *t*1, which corresponds to *T* ¼ 0.

3. Biodistribution

Following the methodology described below, endogenous peroxidases were inhibited using a 1% H_2_O_2_ solution in PBS after the sections were dewaxed and rehydrated for IHC using scDb-hERG1-β1 and scFv-hERG1 antibodies. Proteinase K treatment (5 µg/mL) in PBS at 37°C for 5 minutes was then applied in order to perform antigen retrieval. The sections underwent a 5-minute RT incubation period with an Ultra V Block (LabVision, cat. 12583158) solution. The solution consists of a mixture of ubiquitous proteins necessary to saturate the nonspecific bonds. Incubation with the primary antibodies was carried out overnight at 4°C. Anti-6xHis (1:250) was incubated on the sections for 90 minutes at RT. Then the immunostaining was performed with a commercially available kit (PicTure Max Kit; Invitrogen, cat. 87-9083) according to the manufacturer’s instructions, and nuclei were counterstained with Mayer’s hematoxylin.

4. Kidney perfusion

Kidney perfusion status was assessed using contrast-enhanced US imaging with VevoF2-LAZR-X after intravenous injection of a 50 mL bolus ultrasound (US) contrast agents (Vevo MicroMarker, Bracco Research s.p.a.), equivalent to 2 × 10^7^ bubbles/50 mL bolus. The injection was performed using a 27G butterfly syringe. Fresh dilution of the stock contrast agents was done after each injection. During the procedure, the mouse was placed on the handling table of the imaging platform in the prone position. The first acquisition was performed in B-mode to visualize both kidneys in the same image. A 29 MHz transducer was used for contrast-enhanced US imaging. The data obtained were processed and analyzed using the VevoCQ (Fujifilm VisualSonics).

5. AST/ALT determination

The levels of AST and ALT in mice were measured in blood samples after 24 hours of treatment with scDb-hERG1-β1-TRAIL. Protocol was performed following manufacturer instructions (see the “Reagents” section). The results are reported as the AST/ALT ratio.

6. *In Vivo* Models and pharmacokinetic Studies

Two mouse models of breast cancer have been established. The first was generated by subcutaneous injection of MDA-MB-231 cells on six-week-old female athymic Foxn1 nu/nu mice (Envigo S.r.l, Italy). Before the injection, MDA-MB-231 cells were cultured in DMEM +5% FBS medium under the conditions of 37°C and 5% CO_2_. For the injection, 5 × 10^6^ tumor cell lines were suspended in 100 µL of PBS and injected into the two flanks of mice. To evaluate the therapeutic effect of the scDb-hERG1-β1-TRAIL antibody, 30 days after cell inoculation, mice were divided into two groups of treatment: (i) control (saline; *n* = 4) and (ii) scDb-hERG1-β1-TRAIL (40 mg/kg; *n* = 6). The treatments were administrated intravenously on alternate days starting from day 30 after cell injection and ended on day 37.

To generate the second model, 1 × 10^6^ MDA-MB-231 cells were implanted into the fourth mammary fat pad of six-week-old NOD SCID female mice (Charles River Laboratories). Twenty-six days after cell injection, mice were randomized into two groups of treatment: control (saline; *n* = 7) and scDb-hERG1-β1-TRAIL (8 mg/kg; *n* = 8). The treatments were administrated intravenously on alternate days starting from day 26 after cell injection and ended on day 40.

All the experiments were performed at the Animal House (Ce.S.A.L) of the University of Florence. Mice were housed inside the sterile room in ventilated cabinets with a canonical 12-hour dark–light cycle and unlimited access to food and water. The procedures were performed accomplishing the ARRIVE guidelines for animal research. All the animal experiments received the approval from the Italian Ministry of Health with authorization number 769/2023-PR.

7. US and imaging

In order to assess tumor growth, US imaging was performed with the VevoLAZR-X system (Fujifilm Visualsonics) on live animals. A 55-Mhz transducer was used for the 3D axial scan of tumor masses. The 3D acquisition was performed in B-mode using the 3D motor that allows the transducer to scan the tumor masses in various sections along the longitudinal axis. In each section, the tumor perimeter was bordered (region of interest), and then, by using VevoLAB software, the 3D rendering of the entire tumor masses was generated. During the imaging of tumors, mice were maintained anesthetized by 2% isoflurane on a pad heated at 37°C. scDb-hERG1-β1 and scDb-TRAIL conjugated with ICG (scDb-hERG1-β1-ICG and scDb-TRAIL-ICG, respectively) were administered subcutaneously in the peritumoral region 15 minutes before the imaging session. The photoacoustic (PA) signal of ICG in the tumor was observed by PA imaging (PAI) performed with VevoF2-LAZR-X. PA images were acquired using the 55 MHz linear array transducer. The quantification of the percent of PA signal inside the whole tumor mass was performed by using Vevo Lab software as reported in World Molecular Imaging Congress: September 2023, Prague, oral presentation and September 2024, Montreal, oral presentation.

8. Histology and IHC on tumor masses

Tumor masses of mice were analyzed for anti-His expression (see the detailed protocol in section 3, Biodistribution) and TUNEL assay. After dewaxing and rehydrating, the sections were probed using a commercially available kit (see supplementary reagents section) according to the manufacturer’s instructions.

### Statistical analysis

Unless otherwise indicated, data are given as the means ± SEM in at least three independent experiments. Statistical comparisons were performed with OriginPro 2015 and SAS 9.2 (SAS Institute) software. The normality of data distribution was checked with K-S test. In the case of normal distributions, each data set was first checked for variance homogeneity using the F test for equality of two variances and the Brown–Forsythe test for multiple comparisons. For comparisons between two groups of data, we used the Student *t* test. For multiple comparisons, one-way ANOVA followed by the Bonferroni *post hoc* test was performed to derive *P* values. In case of unequal variances, ANOVA was followed by the Hochberg GT2 *post hoc* method. In the case of nonnormal distributions, nonparametric Kruskal–Wallis ANOVA followed by the Dwass-Steel-Critchlow-Fligner *post hoc* method was applied. The relevant *P* values are reported in the figure legends.

### Data availability

The data generated in this study are available within the article and its supplementary data files. The raw data are available on request from the corresponding author.

## Results

### Generation and target antigen–specific binding of the scDb-hERG1-β1-TRAIL antibody

**Figure 2.**
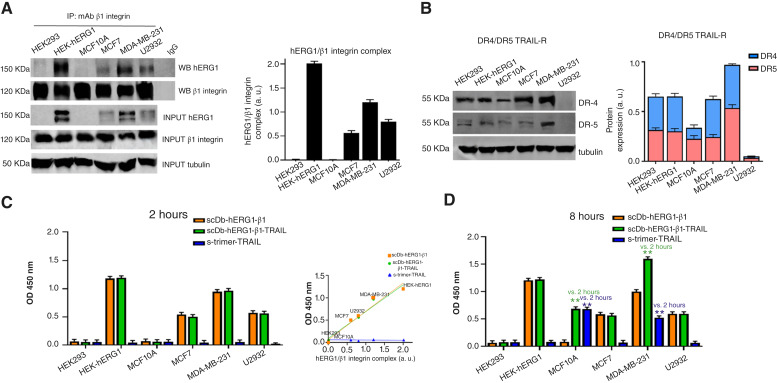
Cell-based ELISA of scDb-hERG1-β1, scDb-hERG1-β1-TRAIL, and s-trimer-TRAIL on cells with different expressions of the hERG1/β1 integrin complex and TRAIL receptors. **A,** Co-immunoprecipitation (IP) between hERG1 and β1 integrin in different cell lines. INPUT, total protein lysate. (Left) Representative WB; (right) densitometric analysis. **B,** WBs of DR4 and DR5 expression in different cell lines. (Left) Representative WB; (right) densitometric analysis. In **A** and **B,** values are expressed as arbitrary units (a.u.) and are means ± SEM of three independent experiments. **C,** Cell-based ELISA using different cells as coating. scDb-hERG1-β1, scDb-hERG1-β1-TRAIL, and s-trimer-TRAIL were incubated for 2 hours at 1.5 µmol/L. Inset: Correlation between the amount of hERG1/β1 integrin complex and OD 450 nm of scDb-hERG1-β1 (PCoC  *R*^2^ = 0.9626, *P* = 0.0005), scDb-hERG1-β1-TRAIL (PCoC, *R*^2^ = 0.9635, *P* = 0.0005), and s-trimer-TRAIL (PCoC *R*^2^ = 0.1125, *P* = 0.5158). **D,** Cell-based ELISA using different cells as coating. scDb-hERG1-β1, scDb-hERG1-β1-TRAIL, and s-trimer-TRAIL were incubated for 8 hours at 1.5 µmol/L. In **C** and **D**, values are expressed as OD 450 nm and are means ± SEM of three independent experiments. Statistics reported in the panel refer to comparisons with values in **C**. **, *P* < 0.01 (one-way ANOVA). OD, optical density; PCoC, Pearson correlation coefficient.

The scDb-hERG1-β1-TRAIL was developed by fusing three TRAIL sequences to the sequence encoding scDb-hERG1-β1 which specifically targets the hERG1/β1 integrin complex in cancer cells (20, 26). Figure 1A shows the flow chart of the developing procedure and the scheme and deduced amino acid sequence of the resulting product. Details are in Supplementary Materials and Methods and Supplementary Fig. S1A–S1E. The chromatograms of the supernatants purified from either GS115-pPI9K-scDb-hERG1-β1 *Pichia pastoris* yeasts or CHO-K1-pSEcTag2A-scDb-hERG1-β1-TRAIL cells show a single peak consistent with the elution of a single protein (Fig. 1B). Coomassie gels and WB show a single band of 68 kDa for scDb-hERG1-β1 (20) and 130 kDa for scDb-hERG1-β1-TRAIL, either in reducing or nonreducing conditions (Fig. 1C). The molecular weight of scDb-hERG1-β1-TRAIL corresponds to the sum of the molecular weights of scDb-hERG1-β1 and of three TRAIL sequences (63 kDa; ref. 27). The binding of scDb-hERG1-β1 and scDb-hERG1-β1-TRAIL to their peptide antigens (listed and described in Supplementary Table S3) was evaluated by ELISA. In particular, the two peptides specific for hERG1 and β1 integrin [S5P and NK12, mixed 1:1 as in (20)] and peptides specific for TRAIL-Rs DR4 (TRL-HM2R1) and DR5 (DR5-HM201; ref. 28) were used. Both scDb-hERG1-β1 and scDb-hERG1-β1-TRAIL exhibited a strong and dose-dependent binding toward the mixture of the hERG1 and β1 peptides (Fig. 1D, see also Supplementary Fig. S1F). scDb-hERG1-β1 did not bind to TRAIL-R–specific peptides (Fig. 1E, see also Supplementary Fig. S1G), whereas scDb-hERG1-β1-TRAIL had a strong and dose-dependent binding to both DR4 and DR5 peptides, similarly to a trimeric soluble form of TRAIL (s-trimer TRAIL; Fig. 1E). A cell-based ELISA was then performed on cells that show variable expressions of either the hERG1/β1 complex (Fig. 2A) or the TRAIL-Rs DR4/DR5 (Fig. 2B). In particular, we used: (i) HEK293 cells (normal human kidney epithelial cells) with no expression of hERG1/β1 and medium expression of DR4/DR5 (29); (ii) HEK-hERG1 cells [HEK 293 cells stably transfected with and hence overexpressing hERG1 (23)] with high expression of hERG1/β1 and medium expression of DR4/DR5 (29); (iii) MCF10A [a benign tumor breast epithelial cell line (30)] with very low expression of hERG1/β1 and medium expression of DR4/DR5 (31); (iv) MCF 7 [a luminal A breast cancer cell line(32)] with medium expression of hERG1/β1 integrin and medium/high expression of DR4/DR5 (33); (v) MDA-MB-231 [a TNBC cell line (34)] with very high expression of hERG1/β1 and high expression of DR4/DR5 (33); and (vi) U2932 (a diffuse large B-cell lymphoma cell line (35) with moderate expression of hERG1/β1 and no expression of either DR4 or DR5. The cell-based ELISA was performed using different molar concentrations of scDb-hERG1-β1, scDb-hERG1-β1-TRAIL, and s-trimer TRAIL and evaluated after either 2 (2 hours) or 8 (8 hours) hours of incubation. The dose–response curves are in Supplementary Fig. S2A, and data relative to the concentration of 1.5 µmol/L are in Fig. 2C (2 hours) and 2D (8 hours), respectively. At 2 hours, both scDb-hERG1-β1 and scDb-hERG1-β1-TRAIL bound to the various cell lines (Fig. 2C) with different strengths which likely depended on the amount of hERG1/β1 integrin expressed by the cells (see the correlation analysis in the inset to Fig. 2C) but not of DR4/DR5 (Supplementary Fig. S2B). The s-trimer TRAIL showed no binding to any of the cell lines analyzed (Fig. 2C) nor had any competitive effect over the binding of either scDb-hERG1-β1 or scDb-hERG1-β1-TRAIL on MDA-MB-231 (Supplementary Fig. S2C). At 8 hours, (i) the binding of scDb-hERG1-β1, scDb-hERG1-β1-TRAIL, and s-trimer TRAIL remained roughly unchanged in HEK293, HEK-hERG1, MCF 7, and U2932 cells (Fig. 2D) and (ii) the binding of scDb-hERG1-β1-TRAIL and s-trimer TRAIL (but not of scDb-hERG1-β1) to MCF10A and MDA-MB-231 cells significantly increased compared with 2 hours (Fig. 2D), with a slight correlation with the expression of DR4/DR5 (Supplementary Fig. S2D).

Overall, the main determinant in the binding capacity of scDb-hERG1-β1-TRAIL is represented by scDb-hERG1-β1, which recognizes the hERG1/β1 integrin complex, whereas TRAIL-Rs are bound more slowly and, in general, with less avidity.

### Effects of scDb-hERG1-β1-TRAIL on breast cancer cell vitality, apoptosis, and proliferation

**Figure 3.**
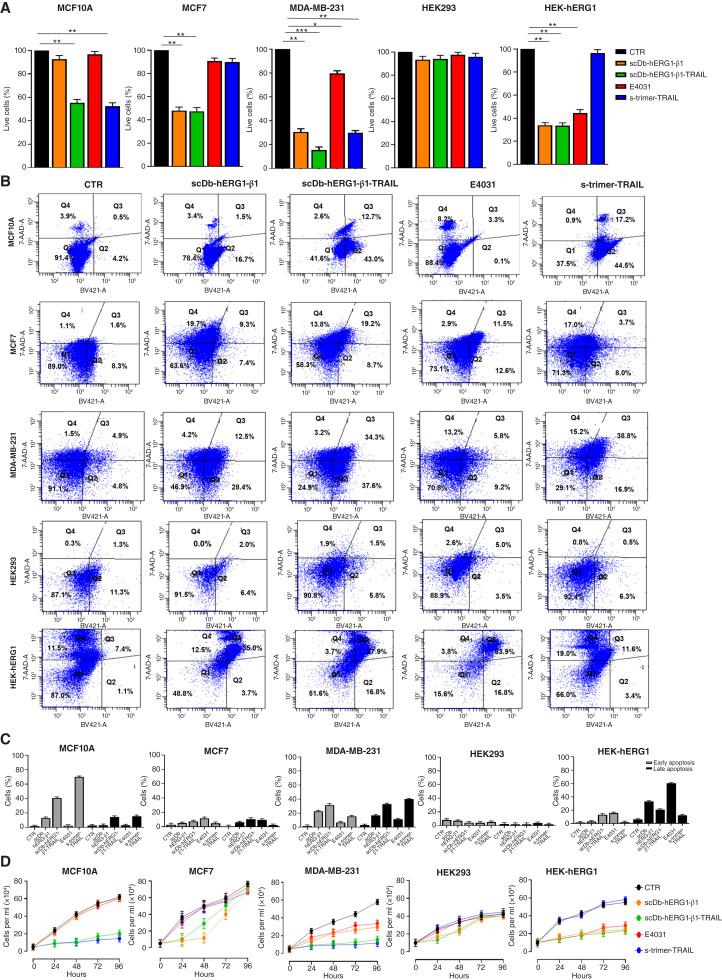
Effects of the scDb-hERG1-β1-TRAIL, scDb-hERG1-β1, s-trimer-TRAIL, and E4031 on cell viability, apoptosis, and proliferation. **A,** Percentage of live cells after 24 hours of treatment with scDb-hERG1-β1-TRAIL, scDb-hERG1-β1, and s-trimer-TRAIL at 0.9 µmol/L and E4031 at 40 µmol/L. Values are means ± SEM of three independent experiments. **B,** Representative dot plots of the annexin V test after 24 hours of treatment as in **A**. The percentages of cells in the different quartiles (Q1, live; Q2, early apoptotic; Q3, late apoptotic; Q4, necrotic) are reported inside each panel. **C,** Bar graph showing the percentage of cells in early and late apoptosis after 24 hours of treatment as in **A**. Values are means ± SEM of three independent experiments. **D,** Number of live (trypan blue negative) cells at different times of treatment as in **A**. Values are means ± SEM of three independent experiments. *, *P* < 0.05; **, *P* < 0.01; ***, *P* < 0.001 (one-way ANOVA). CTR, control (untreated cells).

**Figure 4.**
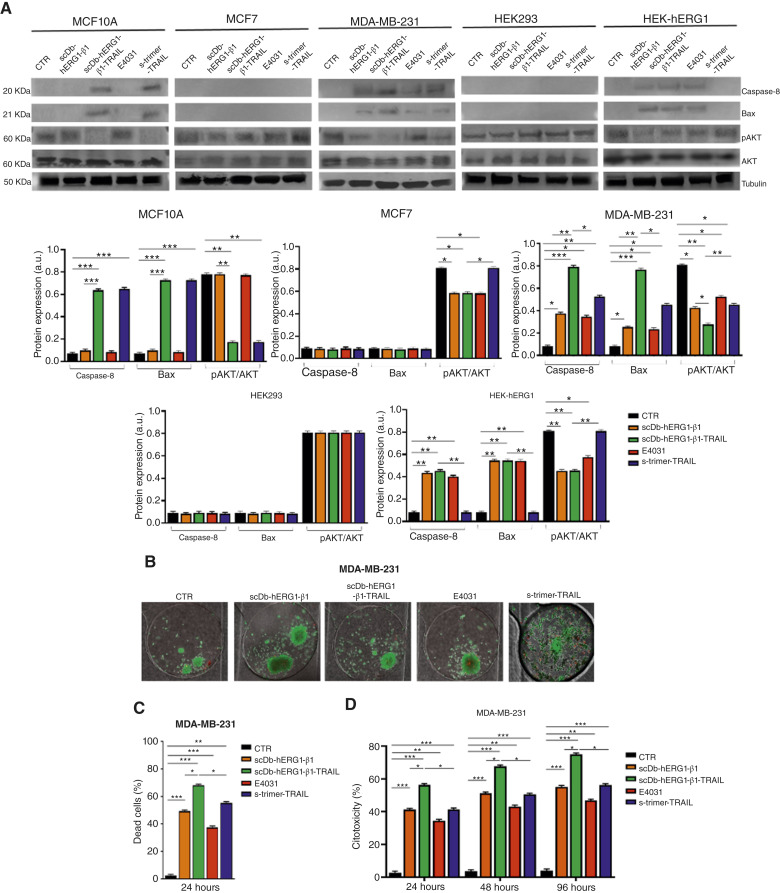
Effects of the scDb-hERG1-β1-TRAIL, scDb-hERG1-β1, s-trimer-TRAIL, and E4031 on cell signaling and vitality in a microfluidic 3D culture system. **A,** Representative blot (top) and densitometric analysis (bottom) of caspase 8, Bax, and pAKT expression in different cell lines treated for 90 minutes as in Fig. 3A. Values are means ± SEM of three independent experiments. **B,** Representative images of tumor aggregates of MDA-MB-231 cells loaded into the microfluidic chip chambers and treated for 24 hours as in Fig. 3A, stained with Calcein/PI (Calcein = green; PI = red). **C,** Bar graph of the mean red fluorescence (dead cells) of MDA-MB-231 cells treated as in Fig. 3A. All values are means ± SEM of three independent experiments. **D,** Bar graph of cytotoxicity (%) calculated through the release of LDH in MDA-MB-231 cells treated as in Fig. 3A for 24, 48, and 96 hours. Values are means ± SEM of three independent experiments. *, *P* < 0.05; **, *P* < 0.01; ***, *P* < 0.001 (one-way ANOVA). a.u., arbitrary units; CTR, control (untreated cells).

The functional effects of scDb-hERG1-β1-TRAIL were then tested and compared with those of scDb-hERG1-β1, of the s-trimer TRAIL and of E4031, a specific blocker of hERG1 currents (36). We used the breast cancer cell lines used in Fig. 2, which are known to have different responses to TRAIL-based treatments: MCF10A and MDA-MB-231 are TRAIL-sensitive (33), whereas MCF 7 is TRAIL-resistant (33). In addition, we used HEK293 and HEK-hERG1 cells, both TRAIL-resistant (29, 31), as controls sbecause they have no or very high expression of the hERG1/β1 integrin complex, respectively. After determining the IC_50_ doses (Supplementary Fig. S3A–S3C; Supplementary Table S4), we chose the dose of 0.9 µmol/L for scDb-hERG1-β1-TRAIL, scDb-hERG1-β1, or s-trimer TRAIL, whereas E4031 was used at 40 µmol/L as in (37). Both cell vitality (using the trypan blue exclusion test) and apoptosis (using the annexin V/PI test) were evaluated after 24 hours of treatment. The scDb-hERG1-β1-TRAIL reduced cell vitality with a progressively greater efficacy in MCF10A, MCF7, and MDA-MB-231 cells. A reduction of cell vitality in HEK-hERG1 cells but not in HEK293 cells was detected. In MCF 10A and MDA-MB231 cells, the activity of scDb-hERG1-β1-TRAIL in decreasing cell vitality was higher compared with scDb-hERG1-β1. E4031 decreased cell viability only in HEK-hERG1 cells and slightly in MDA-MB-231 cells. The s-trimer-TRAIL strongly reduced cell viability in MCF 10A and MDA-MB-231 cells but not in MCF7, HEK293 and HEK-hERG1, as expected (Fig. 3A). Similar effects were observed on cellular apoptosis. Figure 3B shows representative dot blots with the percentage of cells in the different stages. Figure 3C shows the percentage of cells in early and late apoptosis obtained in three different experiments. Indeed, scDb-hERG1-β1-TRAIL induced a relevant apoptosis in MCF10A and MDA-MB 231 cells, higher than that induced by scDb-hERG1-β1. Interestingly, in MDA-MB-231 TNBC cells, the proapoptotic effects of scDb-hERG1-β1-TRAIL were particularly strong and produced an almost complete reduction of cell proliferation (Fig. 3D). These results indicated a relevant functional activity of scDb-hERG1-β1-TRAIL and prompted us to analyze the signaling pathway underlying such effects. scDb-hERG1-β1-TRAIL increased the levels of cleaved (meaning active) caspase-8 and the proapoptotic protein of the BCL family, BAX, mainly in TRAIL-sensitive cells and decreased the phosphorylation of AKT, which inhibits TRAIL-induced apoptosis (38), in all the cell lines except HEK293 cells. These effects were similar to those exerted by s-trimer-TRAIL and were particularly strong in TNBC MDA-MB-231 (Fig. 4A and the related densitometric analysis in the bar graph on the bottom). No effects on the expression of cyclins D and E were observed with any treatment (Supplementary Fig. S4A).

scDb-hERG1-β1-TRAIL (as well as scDb-hERG1-β1, s-trimer-TRAIL, and E4031) was then tested on MDA-MB-231 TNBC cells cultured as 3D within a microfluidic system which better mimics the conditions occurring within the tumor tissue (39). The system (40) is described in the “Materials and Methods” and Supplementary Fig. S4B. After checking the successful loading of the tumor aggregates of MDA-MB-231 cells into the chip through the “tumor inlet” (Fig. 4B), the different treatments (scDb-hERG1-β1-TRAIL, scDb-hERG1-β1, s-trimer TRAIL, and E4031) were added through an approach named “linear withdraw” (Supplementary Fig. S4B), and their effects on cell vitality was determined after 24 hours through the “calcein/PI” assay. The scDb-hERG1-β1-TRAIL induced a higher percentage of dead cells (Fig. 4C). The long-term effects of the treatments were tested through a different perfusion approach called “linear push perfusion.” The perfusing medium was collected every 24 hours, from T_24_ to T_96_, and the amount of LDH was determined as a measure of cytotoxicity. A progressive, time-dependent increase in cytotoxicity was observed with scDb-hERG1-β1-TRAIL, stronger than with the other treatments (Fig. 4D). Overall, all the data shown in Figs. 3 and 4 indicate that scDb-hERG1-β1-TRAIL exerts proapoptotic and cytotoxic effects, which are particularly evident in TNBC MDA-MB-231 cells.

### *In vivo* analysis of the scDb-hERG1-β1-TRAIL antibody

a. Pharmacokinetics and toxicity

**Figure 5.**
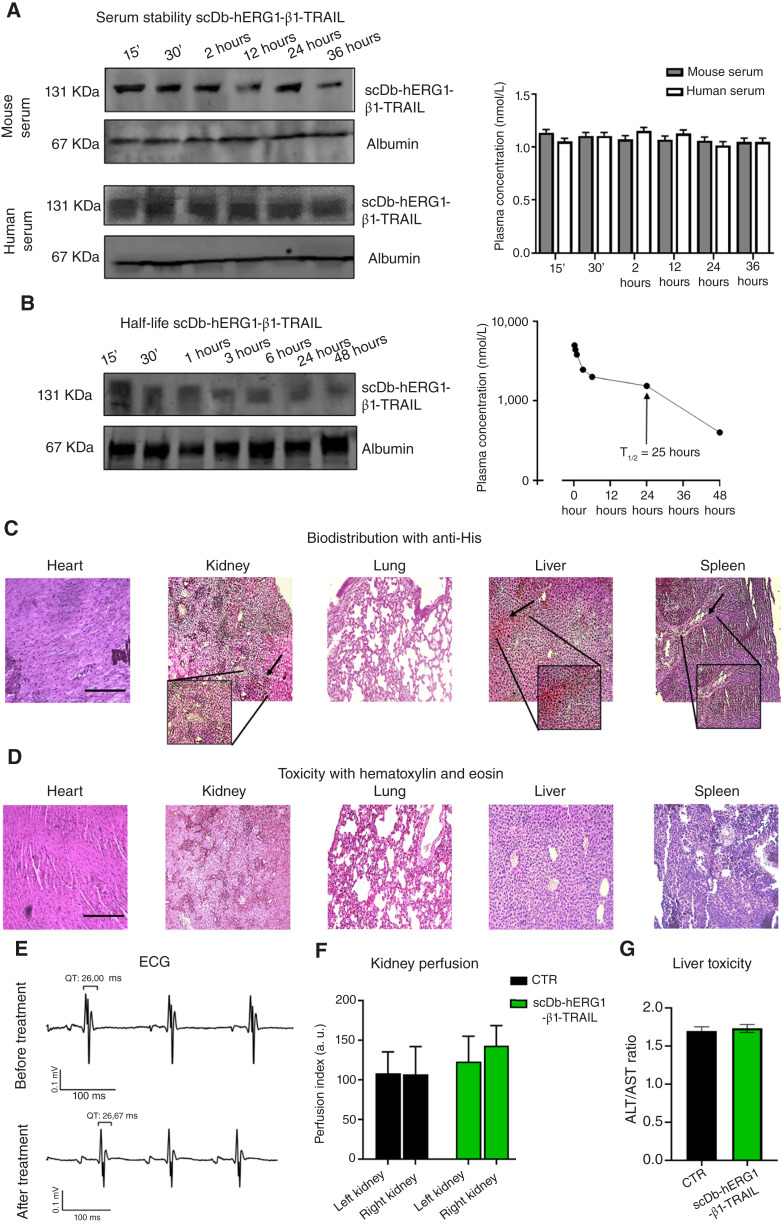
Pharmacokinetic and toxicity *in vivo* of the scDb-hERG1-β1-TRAIL. **A,** Serum stability. (Left) Representative WBs in nonreducing conditions of the scDb-hERG1-β1-TRAIL (150 mg/mL) in mouse (top) and human (bottom) serum after incubation at 37°C for different times. (Right) Densitometric analysis. Values are reported as concentration and are means ± SEM of three independent experiments. **B,***In vivo* half-life. (Left) Representative WB in nonreducing conditions of the scDb-hERG1-β1-TRAIL injected intravenously at 35 mg/kg in FVB mice and present in blood samples collected at different times. (Right) Densitometric analysis. The half-life (T_1/2_) was calculated as detailed in the Supplementary Methods. Values are means ± SEM of three independent experiments. **C,** Biodistribution of the scDb-hERG1-β1-TRAIL injected intravenously as in **B** and evaluated by IHC staining with anti-6xHis antibodies in different organs. Scale bar, 200 μm. **D,** Toxicity of the scDb-hERG1-β1-TRAIL injected intravenously as in **B** and evaluated by H&E staining on different organs of mice treated as in **B**. Scale bar, 200 μm. **E,** ECG, (**F**) kidney perfusion index, and (**G**) ALT/AST ratio in serum of mice before and after treatment with scDb-hERG1-β1-TRAIL administered as in **B**. Values are means ± SEM of three independent experiments. CTR, control.

scDb-hERG1-β1-TRAIL was then tested *in vivo*, first determining its pharmacokinetic and toxicologic profile. First, we measured its stability against proteolytic activities contained in serum, either mice or human. The WBs on gels run in nonreducing conditions showed that scDb-hERG1-β1-TRAIL was present after 96 hours of incubation at 37°C either in mouse or human serum, with no evident sign of degradation (Fig. 5A and the bar graph on the right showing plasma concentration values; WBs from gels in reducing conditions are in Supplementary Fig. S5A). We then determined the pharmacokinetic characteristics of the scDb-hERG1-β1-TRAIL antibody injected intravenously into FVB mice at the dose of 35 mg/kg, i.e., a dose comparable with that used for scDb-hERG1-β1 [16 mg/kg (20, 22)]. The plasma concentrations of scDb-hERG1-β1-TRAIL determined by WB at different time points [Fig. 5B (left)] showed a characteristic two-phase pharmacokinetic behavior, with a rapid distribution phase and a longer elimination phase. The half-life of the elimination phase turned out to be 25 hours [Fig. 5B (right); Supplementary Fig. S5B]. The accumulation of the injected scDb-hERG1-β1-TRAIL in internal organs (heart, liver, kidney, spleen, and lung) was determined on tissue samples collected 3 hours after injection by IHC using the anti-6xHis antibody. scDb-hERG1-β1-TRAIL did not accumulate in the heart and lung and slightly accumulated in the liver (20%), glomerular area of the kidneys (40%), and spleen (10%; Fig. 5C, the site of accumulation is highlighted in the insets, in Supplementary Fig. S5C, control (CTR) mice treated with vehicle are reported). No gross morphologic alterations evaluated by hematoxylin and eosin staining were observed in all the tested organs (Fig. 5D Hematoxylin and eosin staining of untreated mice are reported in Supplementary Fig. S5D for comparison). Consistently, no changes in ECG (Fig. 5E), renal perfusion (Fig. 5F), or levels of liver markers (AST and ALT) in blood samples (Fig. 5G) were observed in mice treated with scDb-hERG1-β1-TRAIL compared with untreated animals.

b. Pharmacodynamics in TNBC mouse models

**Figure 6.**
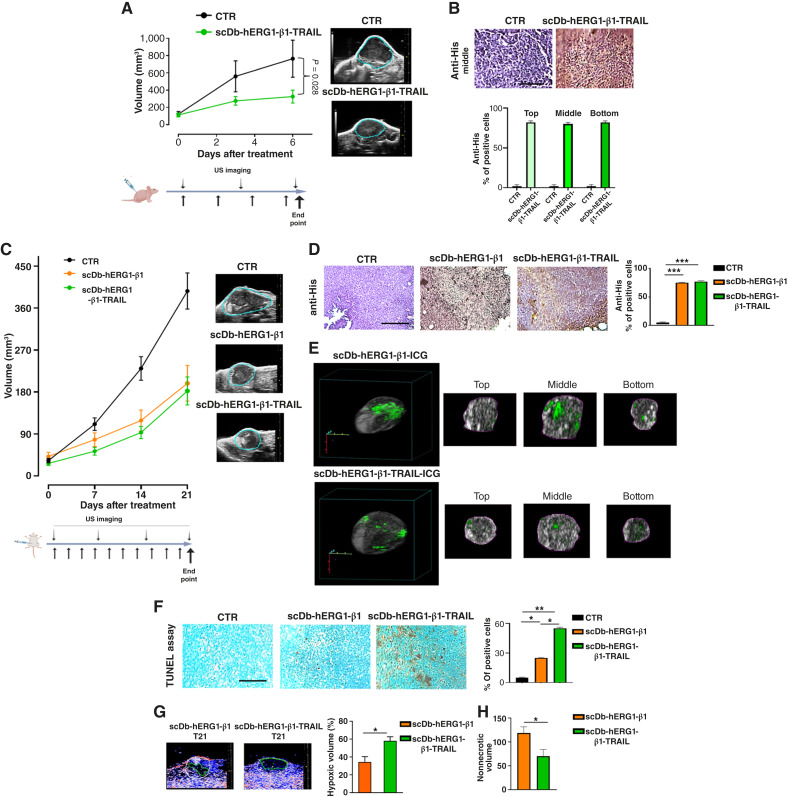
Pharmacodynamics *in vivo* of the scDb-hERG1-β1-TRAIL on triple-negative breast cancer models. **A,** Time course (upper) of the volume of tumor masses arising from subcutaneous injection of MDA-MB-231 cells into athymic nude mice, untreated and treated with scDb-hERG1-β1-TRAIL administered intravenously at 35 mg/kg, following the schedule shown in the panel on the bottom. (Right) Representative US images of tumor masses at the endpoint. **B,** Representative images (top) and bar graph quantification (bottom) of IHC staining with anti-6xHis antibodies of tumor masses. Scale bar, 200 µm. **C,** Time course (upper) of the volume of tumor masses arising from orthotopic injection of MDA-MB-231 cells in the fat pad of NOD-SCID mice, untreated (CTR) and treated with scDb-hERG1-β1 (orange symbols) and scDb-hERG1-β1-TRAIL (green symbols) administered intravenously at 16 or 35 mg/kg, respectively, following the schedule shown in the panel on the bottom. (Right) Representative US images of tumor masses at the endpoint. **D,** Representative images (images on the left) and bar graph quantification (panel on the right) of IHC staining with anti-6xHis antibodies of tumor masses collected at the endpoint. Scale bar, 200 µm. **E,** Representative images of scDb-hERG1-β1 and scDb-hERG1-β1-TRAIL conjugated with ICG in tumor masses from untreated mice. Scale bar, 200 µm. **F,** Representative images (left) and bar graph quantification (right) of TUNEL staining of apoptotic cells in masses collected at the endpoint from mice treated as in **C**. **G,** Representative photoacoustic images (left) and bar graph quantification (right) of masses from mice treated as in **C** at the endpoint. The hypoxic volume (%) calculated as detailed in “Materials and Methods” is shown. **H,** Bar graph of the non-necrotic volume of tumor masses from mice treated as in **C** at the endpoint. *, *P* < 0.05; **, *P* < 0.01; ***, *P* < 0.001. CTR, control.

The *in vivo* antineoplastic activity of scDb-hERG1-β1-TRAIL was then evaluated in 2 mouse models of TNBC: (i) a subcutaneous xenograft in immunodeficient nu/nu mice (Fig. 6A) and (ii) an orthotopic xenograft in the fourth mammary fat pad of NOD SCID mice (Fig. 6C). In either models, MDA-MB-231 cells were used and tumor growth was monitored by high-frequency US. Based on its half-life (25 hours), scDb-hERG1-β1-TRAIL was administered intravenously every 48 hours for either 7 (model 1) or 21 days (model 2) at the dose of 35 mg/kg, starting when the tumor masses reached a volume of 180 mm^3^ (model 1) or 30 mm^3^ (model 2; see the schemes on the bottoms of Fig. 6A and C). In model 2, mice were also treated with scDb-hERG1-β1, administered intravenously at 16 mg/kg daily for 21 days (20).

scDb-hERG1-β1-TRAIL significantly reduced the volume of subcutaneous masses arising from MDA-MB-231 cells after 1 week of treatment (Fig. 6A). The antibody was able to penetrate within the tumor masses, as evidenced by the IHC staining with anti-His antibodies at top, medium, and bottom levels of the masses (Fig. 6B). The treatment with scDb-hERG1-β1-TRAIL produced a statistically significant reduction of the tumor masses growing in the mammary fat pad of NOD SCID mice after orthotopic injection of MDA-MB-231 cells, which was comparable with that produced by scDb-hERG1-β1 (Fig. 6C). Both scDb-hERG1-β1 and scDb-hERG1-β1-TRAIL penetrated into tumor masses as shown by (i) IHC staining with anti-His antibodies of the tumor masses collected at the end of the experiment (Fig. 6D) and (ii) photoacoustic imaging (PAI) using scDb-hERG1-β1 and scDb-hERG1-β1-TRAIL conjugated with ICG and injected locally *in vivo* (Fig. 6E). Apoptosis was evaluated in tumor masses collected at the end of treatments through the TUNEL assay (41). A significantly higher percentage of apoptotic (TUNEL-positive) cells was evident in tumor masses from mice treated with scDb-hERG1-β1-TRAIL compared with scDb-hERG1-β1 (Fig. 6F). This effect was mirrored by a significantly higher hypoxic volume determined in living animals by PAI (Fig. 6G) and by larger necrotic areas at necroscopy (Supplementary Fig. S6D). When considering only the nonhypoxic (i.e., the nonnecrotic) volume, tumor masses from mice treated with scDb-hERG1-β1-TRAIL had a volume of roughly 70 mm^3^, meaning a roughly complete arrest in tumor growth, with an effect significantly stronger than in tumor masses from mice treated with scDb-hERG1-β1 (Fig. 6H). All the details and original pictures are in “Materials and Methods” and Supplementary Fig. S6A–S6D.

## Discussion

In the present article, we describe a novel recombinant, single-chain antibody which targets the proteins hERG1, β1 integrin, and TRAIL-Rs on the plasma membrane of cancer cells. Such “trispecific” antibody, named scDb-hERG1-β1-TRAIL, derives from the fusion of three TRAIL sequences to an existing diabody (scDb-hERG1-β1) which binds to the complex formed by hERG1 and β1 integrin. It combines the specific targeting of cancer cells by the scDb-hERG1-β1 with the proapoptotic activity triggered by TRAIL. Indeed, scDb-hERG1-β1-TRAIL exerts proapoptotic, antiproliferative effects in breast cancer cells, mainly TNBC, which express the hERG1/β1 integrin complex and are TRAIL-sensitive, cultured *in vitro* either in classic 2D or 3D cultures within a microfluidic chip. Furthermore, scDb-hERG1-β1-TRAIL showed a good pharmacokinetic and toxicologic profile *in vivo*, accumulated into tumor masses arising from TNBC cells and strongly reducing their growth by inducing apoptotic death.

The scDb-hERG1-β1-TRAIL antibody is easily produced in CHO cells, stable at 4°C, and secreted as a monomer, a not commonly occurring positive feature (42). scDb-hERG1-β1-TRAIL binds efficiently to the peptides recognized by scDb-hERG1-β1 as well as to TRAIL-R–specific peptides (28). Its binding avidity to live cells is mainly determined by the scDb-hERG1-β1 component, whereas only longer incubations are needed to obtain a good binding to cells in dependence on their expression of DR4/DR5 TRAIL-Rs. This occurs also with a soluble trimeric form of TRAIL and could be related to a lower binding avidity of the TRAIL sequence for its receptor(s) (43) or to a time-dependent conformational change in scDb-hERG1-β1-TRAIL which allows a better recognition of the TRAIL-Rs (44). Overall, the targeting moiety of scDb-hERG1-β1-TRAIL is carried by the diabody scDb-hERG1-β1, whereas the proapoptotic effects depend on the activation of TRAIL-R–dependent pathways involving caspase-8 and Bax. Thanks to this double moiety, scDb-hERG1-β1-TRAIL shows a high specificity for cancer cells which often represents a hindrance in TRAIL-related therapeutic approaches (15, 45). The proapoptotic and antiproliferative effects of scDb-hERG1-β1-TRAIL were observed in breast cancer cells, mainly TRAIL-sensitive TNBC cells, and were higher than those exerted by scDb-hERG1-β1, a soluble TRAIL trimer, and the classic hERG1 blocker E4031. The efficacy of scDb-hERG1-β1-TRAIL on TNBC cells was also demonstrated in 3D cell cultures using a novel microfluidic system which represents a pivotal platform for personalized medicine (40).

scDb-hERG1-β1-TRAIL showed a good penetration into cancer tissues *in vivo*, hence overcoming one of the hindrances (low delivery and nonhomogeneous distribution) for TRAIL linked to full-length antibodies (46, 47), as well as no toxicity to the heart, kidney, and liver. In addition, it had a half-life of 25 hours, much longer compared with that of soluble TRAILs [i.e., minutes (48)] which represents a favorable pharmacodynamic feature for cancer therapeutics (22). Finally, applying up-to-date technologies combining ultrahigh-frequency US and PAI, we showed that scDb-hERG1-β1-TRAIL has a good antineoplastic efficacy in TNBC mouse models, producing a strong reduction in tumor growth with the induction of cell death with the occurrence of intratumoral necrosis which occurs even in relatively small tumor masses.

Overall, we have here provided the proof of concept of the therapeutic valence of a novel scDb-hERG1-β1-TRAIL antibody, which targets the hERG1/β1 integrin complex as well as TRAIL-Rs in breast cancer and TNBC which overexpress the complex, hence representing a novel antibody–drug conjugate for difficult-to-treat cancers.

## Supplementary Material

Supplementary Figure S1 Development of the scDb-hERG1-β1-TRAIL

Supplementary Figure S2 Dose response curves and antibody correlation in different cell lines.

Supplementary Figure S3 Dose-dependence curves of HEK293, HEK-hERG1, MCF10A, MCF7, MDA-MB-231 and U2932

Supplementary Figure S4 Cyclin western-blot and schematic representation of the tumor-on-chip model.

Supplementary Figure S5 Stability and pharmacokinetic data.

Supplementary Figure S6 In vivo data.

Supplementary Table S1 PRODUCTION OF scDb-TRAIL FUSION PROTEIN

Supplementary Table S2 Cycle steps

Supplementary Table S3 Peptides used for ELISA assays.

Supplementary Table S4 IC50 values of the scDb-hERG1-β1, scDb-hERG1-β1-TRAIL and s-trimer-TRAIL on cell viability in different cell lines.
