# Supplementary material for: Antineoplastic Activity of a Novel Trispecific Single-Chain Antibody Targeting the hERG1/β1 Integrin Complex and TRAIL Receptors
Source: Mol Cancer Ther. 2025 Jun 18;24(10):1584–99. doi: 10.1158/1535-7163.MCT-24-0646 (PMC12485380; doi:10.1158/1535-7163.MCT-24-0646)
Supplement: Supplementary Figure S1 — Development of the scDb-hERG1-β1-TRAIL [file mct-24-0646_supplementary_figure_s1_supps1.pdf]

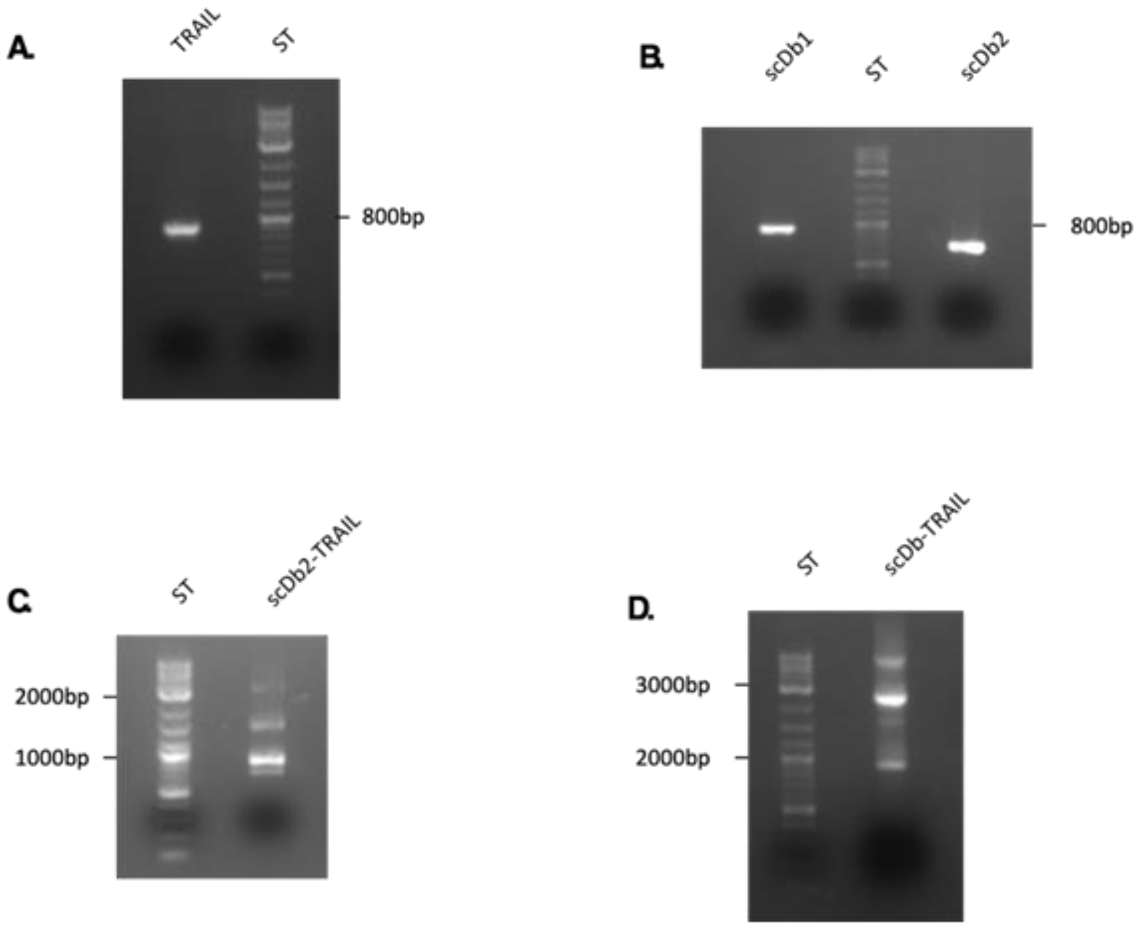

E.

AGC TTG GAG GTC CAA CTG CAA CAG TCT GGA CCT GAA CTG GTG AAG CCT GGG GCT TCT GTG AAG ATA TCC TGC AAG ACT TCA GGA TAC ACA TTC ACT GAA TAC ACC GTT CAC TGG GTG AAA CAG AGC CAT GGA AAG AGC CTT GAA TGG ATT GGA GGC ATT AAT CCT AAT GGT GGT ACT ACC TAT AAT CAG AAG TTC AAG GGC AAG GCC ACA TTG ACT ATT GAC AAG TCC TCC AGC TCA GCC TTC ATG GAG CTC CGC AGC CTG ACA TCT GAG GAT TCT GCA GTC TAT TAC TTT GCA ACA GGT TGG GGA CCT GAC TAC TGG GGC CAA GGC ACC ACT CTC ACA GTC TCC TCA GCC AAA ACA ACA CCC CCA TCA GTC TAT CCA CTG GCC CCT **GGC TCG AGT** GAT ATT GTG ATG ACA CAG ACT CCA ACC ACC ATG GCT GCA TCT CCC GGG GAC AAG ATC ACT ATC ACC TGC AGT GTC AGT TCA ATT ATA AGT TCC AAT TAC CTG CAT TGG TAT AGT CAG AAG CCA GGA TTC TCC CCT AAA CTC TTG

ATT TAT AGG ACA TCC AAT CTG GCT TCT GGA GTC CCA CCT CGC TTC AGT GGC AGT GGG TCT GGG ACC TCT TAC TCT CTC ACA ATT GGC ACC ATG GAG GCT GAA GAT GTT GCC ACT TAC TAC TGC CAG CAG GGT TCT GAT ATT CCA CTC ACG TTC GGT GAT GGG ACC AAG CTG GAC CTG AAA CGG GCT GAT GCT GCA CCA ACT GTA TCC **GGT GGT GGT GGT TCT GGT GGT GGT GGT TCT GGC GGC GGC GGC TCC GGT GGT GGT GGA TCC** GAG GTG AAG GTG GTG GAA TCT GGG GGA GGC TTA GTG AAG CCT GGA GGG TCC CTG AAA CTC TCC TGT GCA GCC TCT GGA TTC ACT TTC AGT AGC TAT ACC ATG TCT TGG GTT CGC CAG ACT CCG GAG AAG AGG CTG GAG TGG GTC GCA ACC ATA AGT AGT GGT GGT TCT TAC ACC TAC TAT CCA GAC AGT GTG AAG GGC CGA TTC ACC ATT TCC AGA GAC AAA GCC AAG AAC ACC CTG TAT TTG CAA ATG GGC AGT CTG AAG TCT GAG GAC ACA GCC ATG TAT TAC TGT ACA AGA ATA GGT TAC GAC GAA GAT TAT GCT ATG GAC CAC TGG GGT CAA GGA ACC TCA GTC ACC GTC TCC TCA GCC AAA ACG ACA CCC CCA TCT GTC TAT **AGT GCA CTG** GAT ATT GTG CTG ACA CAA TCT CCA CTC ACT TTG TCG GTT AAC ATT GGT CAA CCA GCC TCT ATC TCT TGC AAG TCA AGT CAG AGC CTC TTA TAT ACT AAT GGA AAA ACC TAT TTT AAT TGG TTA TTA CAG AGG CCA GGC CAG TCT CCA AAG CGC CTA ATC TAT CTG GTG TCT AAA CTG GAC TCT GGA GTC CCT GAC AGG TTC ACT GGC AGT GGA TCA GGA ACA GAT TTT ACA CTG AAA ATC AGC AGA GTG GAG GCT GAA GAT TTG GGA GTT TAT TAC TGC GCG CAA GGT ACA CAT TTT CCG TGG ACG TTC GGT GGA GGG ACC AAG CTG GAA ATC AAA CGG GCT GAT GCT GCA CCA ACT GTA TCC **GGA GGA GGA GGA AGT GGA GGA GGA GGA AGT GGC GGC GGC GGC TCT**ATGGCCATGATGGAGGTCCAGGGGGGACCCAGCCTGGGACAGACCTGCGTGCTGAT
CGTGATCTTCACAGTGCTCCTGCAGTCTCTCTGTGTGGCTGTAACTTACGTGTACTTTACC
AACGAGCTGAAGCAGATGCAGGACAAGTACTCCAAAAGTGGCATTGCTTGTTTCTTAAA
AGAAGATGACAGTTATTGGGACCCCAATGACGAAGAGAGTATGAACAGCCCCTGCTGGC
AAGTCAAGTGGCAACTCCGTCAGCTCGTTAGAAAGATGATTTTGAGAACCTCTGAGGAA
ACCATTTCTACAGTTCAAGAAAAGCAACAAAATATTTCTCCCCTAGTGAGAGAAAGAGG
TCCTCAGAGAGTAGCAGCTCACATAACTGGGACCAGAGGAAGAAGCAACACATTGTCTT
CTCCAAACTCCAAGAATGAAAAGGCTCTGGGCCGCAAAATAAACTCCTGGGAATCATCA
AGGAGTGGGCATTCATTCCTGAGCAACTTGCACTTGAGGAATGGTGAACTGGTCATCCAT
GAAAAAGGGTTTTACTACATCTATTCCCAAACATACTTTCGATTTCAGGAGGAAATAAAA
GAAAACACAAAGAACGACAAACAAATGGTCCAATATATTTACAAATACACAAGTTATCC
TGACCCTATATTGTTGATGAAAAGTGCTAGAAATAGTTGTTGGTCTAAAGATGCAGAATA
TGGACTCTATTCCATCTATCAAGGGGGAATATTTGAGCTTAAGGAAAATGACAGAATTTT
TGTTTCTGTAACAAATGAGCACTTGATAGACATGGACCATGAAGCCAGTTTTTTCGGGGC
CTTTTTAGTTGGCTAACCTCGA

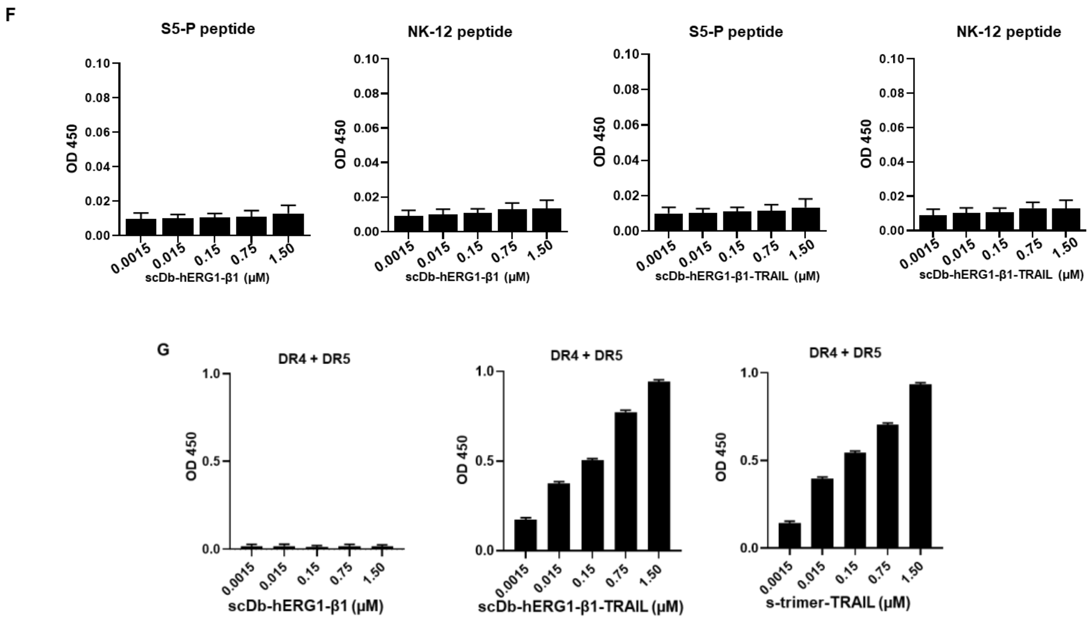

Supplementary Figure S1. Development of the scDb-hERG1-β1-TRAIL. A) isolation of TRAIL; **B)** isolation of scDb1 and scDb2 (NB: scDb 2 is a bit longer than scDb 1. It agrees with Ts2/16 primers location); **C)** scDb2-TRAIL PCR product; **D)** final scDb-TRAIL PCR product. **E)** Final scDb-hERG1-β1-TRAIL. Grey: scDb-hERG1-β1; Yellow: TRAIL; White: linker. **F)** Peptide ELISA using hERG1 S5-P peptide (hERG1) and NK-12 peptide (β1 integrin) as coating antigen with different concentrations of scDb-hERG1-β1 and scDb-hERG1-β1-TRAIL. **G)** Peptide ELISA using DR4 and DR5 peptides as coating antigen with different concentrations of scDb-hERG1-β1, scDb-hERG1-β1-TRAIL and s-trimer-TRAIL. All the values are expressed as OD 450 nm and are means ± SEM of three independent experiments. mAu = milli-Absorbance unit.
