# Supplementary material for: Antineoplastic Activity of a Novel Trispecific Single-Chain Antibody Targeting the hERG1/β1 Integrin Complex and TRAIL Receptors
Source: Mol Cancer Ther. 2025 Jun 18;24(10):1584–99. doi: 10.1158/1535-7163.MCT-24-0646 (PMC12485380; doi:10.1158/1535-7163.MCT-24-0646)
Supplement: Supplementary Figure S2 — Dose response curves and antibody correlation in different cell lines. [file mct-24-0646_supplementary_figure_s2_supps2.pdf]

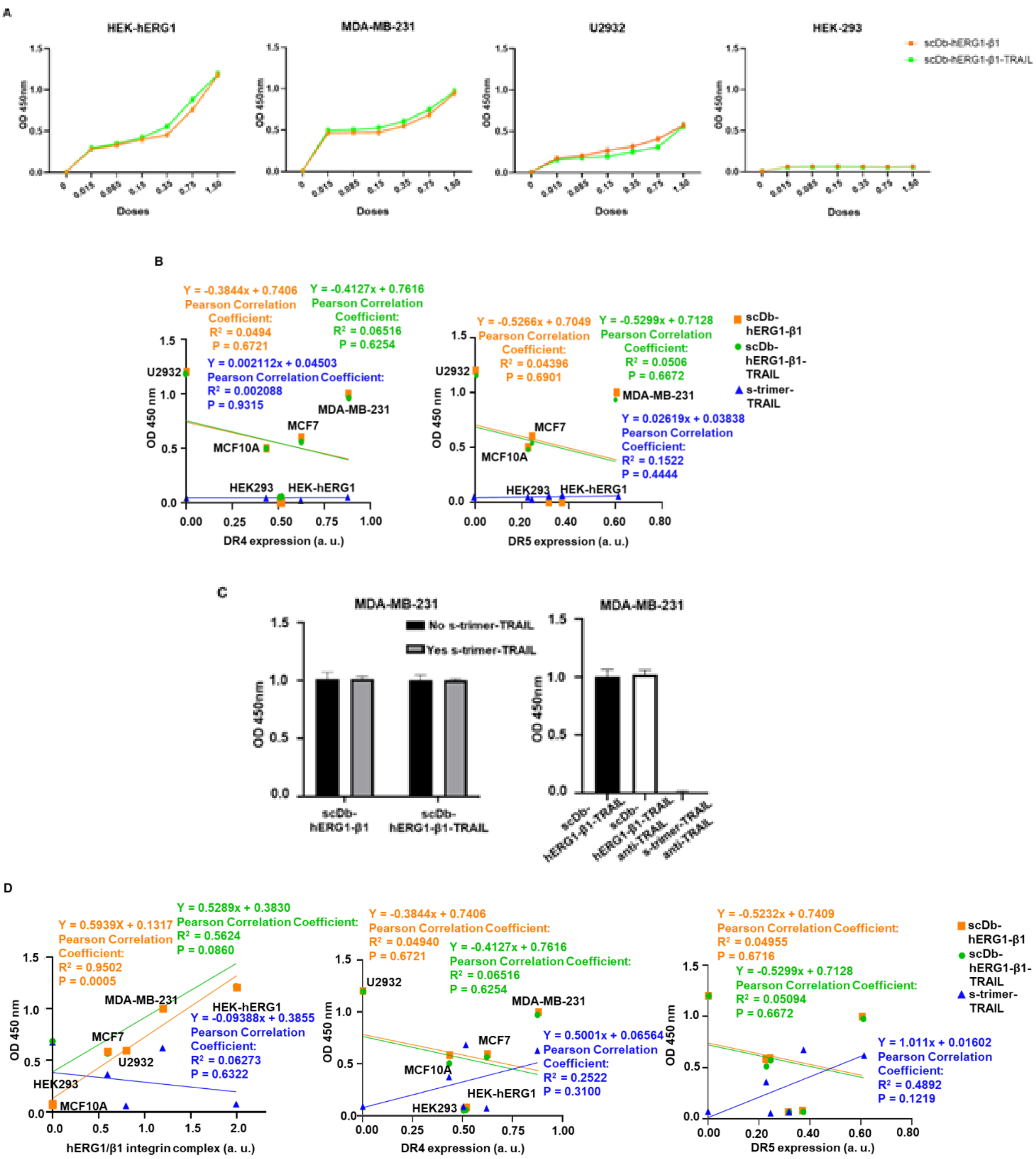

Supplementary Figure S2. Dose response curves and antibody correlation in different cell lines. A) Dose-response curves of the two antibodies in all the different cell lines. **B)** Correlation between DR4 expression and OD 450 nm (left panel) and between DR5 expression and OD 450 nm (right panel) after 2 hours in HEK293, HEK-hERG1, MCF10A, MCF 7, MDA-MB-231 and U2932 cells. **C)** Competitive cell ELISA on MDA-MB-231 cells. MDA-MB-231 were seeded in the presence or in the absence of TRAIL (indicated as "s-trimer TRAIL" and "NO s-trimer TRAIL", respectively), incubated with either scDb-hERG1-β1 (1.5 μM) and scDb-hERG1-β1-TRAIL (1.5 μM) then revealed by anti-His antibody. **D)** Correlation between the hERG1/β1 complex and OD 450nm (left panel), DR4 expression and OD 450 nm

(central panel) and between DR5 expression and OD 450 nm (right panel) after 8 hours in HEK293, HEK-hERG1, MCF10A, MCF 7, MDA-MB-231 and U2932 cells. All the values are expressed as OD 450 nm and are means ± SEM of three independent experiments.
