# Supplementary material for: Antineoplastic Activity of a Novel Trispecific Single-Chain Antibody Targeting the hERG1/β1 Integrin Complex and TRAIL Receptors
Source: Mol Cancer Ther. 2025 Jun 18;24(10):1584–99. doi: 10.1158/1535-7163.MCT-24-0646 (PMC12485380; doi:10.1158/1535-7163.MCT-24-0646)
Supplement: Supplementary Figure S3 — Dose-dependence curves of HEK293, HEK-hERG1, MCF10A, MCF7, MDA-MB-231 and U2932 [file mct-24-0646_supplementary_figure_s3_supps3.pdf]

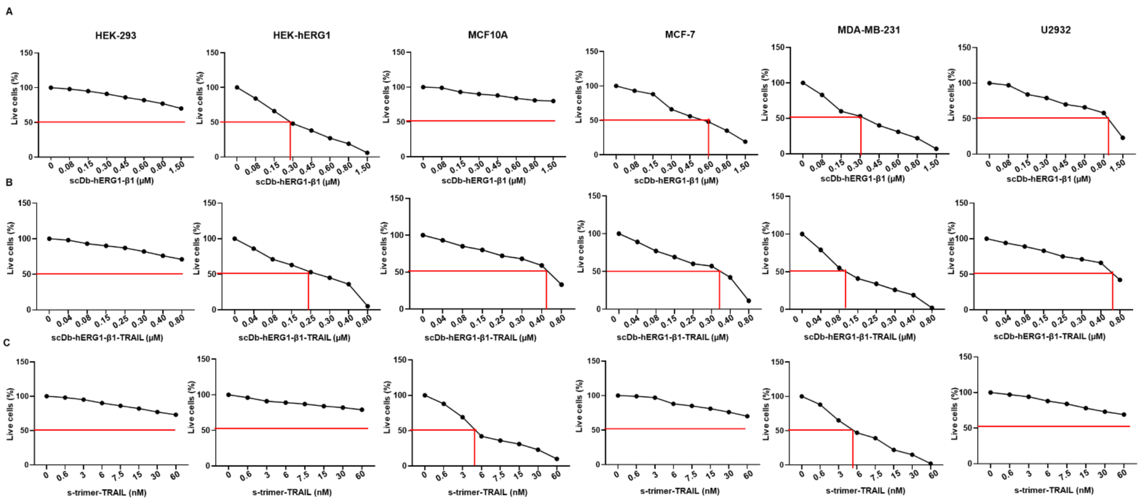

Supplementary Figure S3. Dose-dependence curves of HEK293, HEK-hERG1, MCF10A, MCF7, MDA-MB-231 and U2932. Cells are treated with different concentrations of **A)** scDb-hERG1-β1-TRAIL, **B)** scDb-hERG1-β1, **C)** **s**-trimer-TRAIL, for 24 hours. Red line shows the IC50 value.
