# Supplementary material for: Antineoplastic Activity of a Novel Trispecific Single-Chain Antibody Targeting the hERG1/β1 Integrin Complex and TRAIL Receptors
Source: Mol Cancer Ther. 2025 Jun 18;24(10):1584–99. doi: 10.1158/1535-7163.MCT-24-0646 (PMC12485380; doi:10.1158/1535-7163.MCT-24-0646)
Supplement: Supplementary Figure S4 — Cyclin western-blot and schematic representation of the tumor-on-chip model. [file mct-24-0646_supplementary_figure_s4_supps4.pdf]

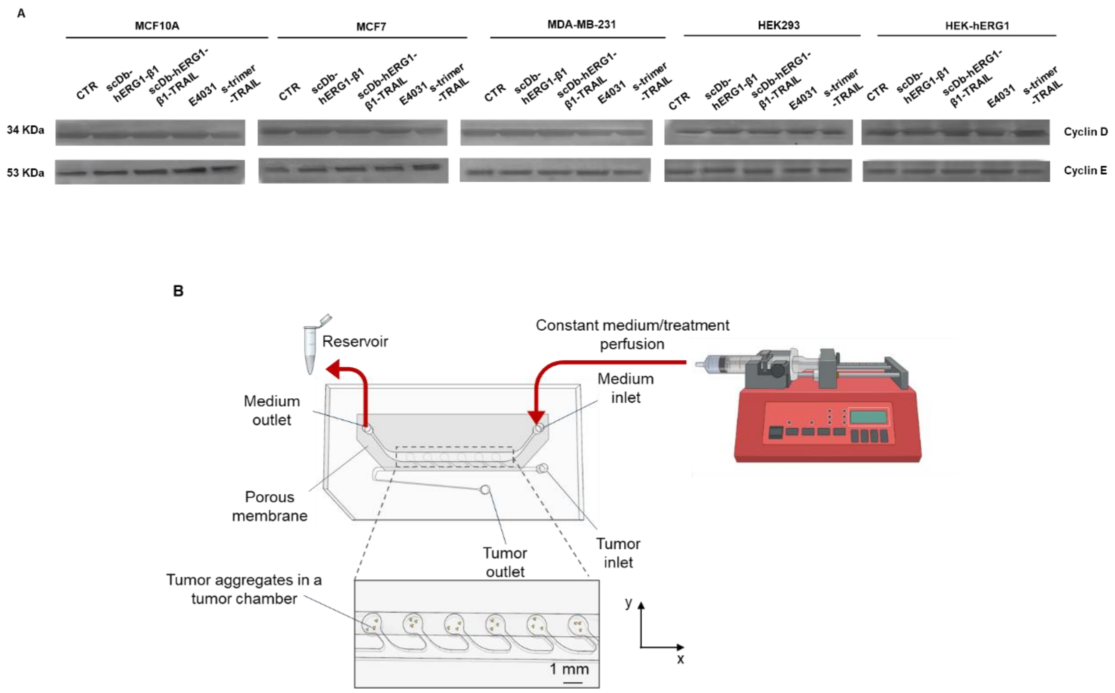

Supplementary Figure S4. Cyclin western-blot and schematic representation of the tumor-on-chip model. A) Representative blot of Cyclin D and Cyclin E expression in MCF10A, MCF 7, MDA-MB-231, HEK293 and HEK-hERG1 cells untreated (CTR) and treated with scDb-hERG1-β1 (0.9 µM), scDb-hERG1-β1-TRAIL (0.9 µM), E4031 (40 µM) and s-trimer-TRAIL (1 µM) for 90 minutes. The corresponding Tubulin is reported in Figure 4A **B)** Schematic representation of the tumor-on-chip model. Tumor aggregates were embedded in a dextran-based hydrogel and cultured in the cylindrical tumor chambers of the chip, with six chambers in total per chip (visual check by microscopy, ECHO Rebel). The tumor chambers are located underneath a medium channel that is separated by a porous membrane. scDb-hERG1-β1-TRAIL treatment was applied through the medium channel via constant linear perfusion.
