# Supplementary material for: Antineoplastic Activity of a Novel Trispecific Single-Chain Antibody Targeting the hERG1/β1 Integrin Complex and TRAIL Receptors
Source: Mol Cancer Ther. 2025 Jun 18;24(10):1584–99. doi: 10.1158/1535-7163.MCT-24-0646 (PMC12485380; doi:10.1158/1535-7163.MCT-24-0646)
Supplement: Supplementary Figure S5 — Stability and pharmacokinetic data. [file mct-24-0646_supplementary_figure_s5_supps5.pdf]

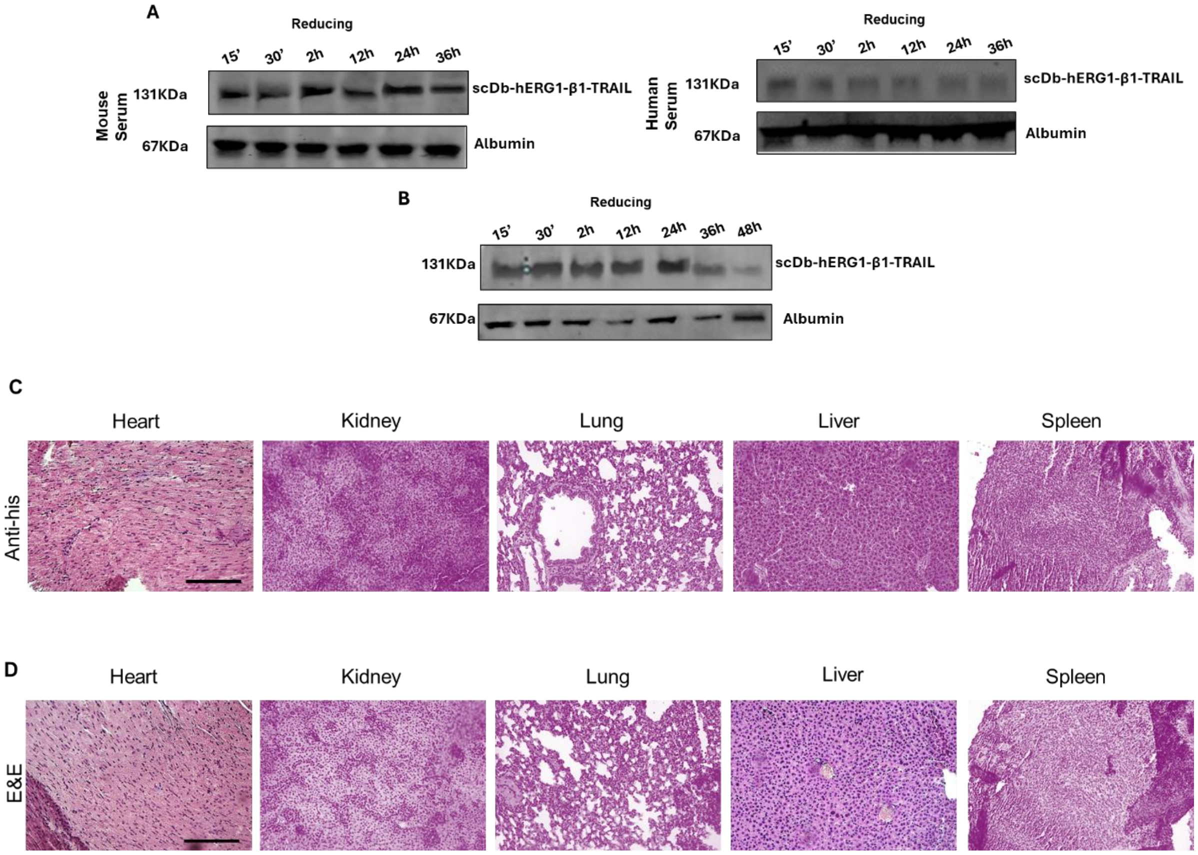

Supplementary Figure S5. Stability and pharmacokinetic data. A) Western blot showing serum stability of the scDb-hERG1-β1-TRAIL (150 mg/mL) in mouse at 37°C at different time points (15', 30', 2h, 12h, 24h, 36h) at reducing conditions. **B)** Western blot half-life at reducing conditions *in vivo* of scDb-hERG1-β1-TRAIL injected intravenously at 8 mg/kg in FVB mice. Blood samples were collected after 15', 30', 1h, 3h, 6h, 24h, and 48h. In **A**, and **B** values are expressed as a.u. (aribitrary units) and are means ± SEM of three independent experiments. **C)** IHC staining with anti-6xHis antibodies of heart, liver, kidney, spleen and lung of mice treated with vehicle (CTR). Scale bar, 200 mm. **D)** H&E staining on organs of mice treated as in B for 24 hours. Scale bar, 200 mm.
