# Supplementary material for: Antineoplastic Activity of a Novel Trispecific Single-Chain Antibody Targeting the hERG1/β1 Integrin Complex and TRAIL Receptors
Source: Mol Cancer Ther. 2025 Jun 18;24(10):1584–99. doi: 10.1158/1535-7163.MCT-24-0646 (PMC12485380; doi:10.1158/1535-7163.MCT-24-0646)
Supplement: Supplementary Figure S6 — In vivo data. [file mct-24-0646_supplementary_figure_s6_supps6.pdf]

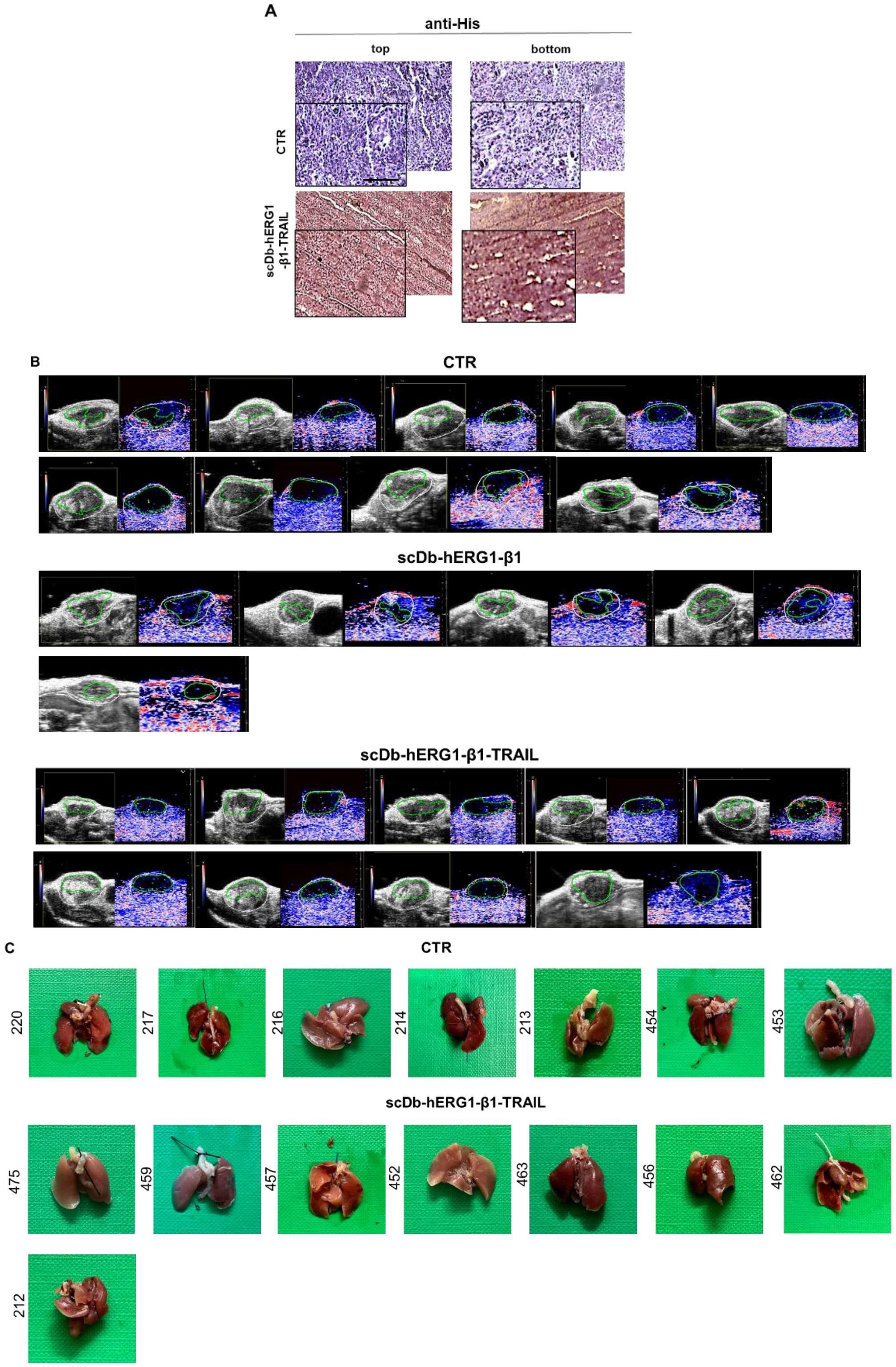
A
anti-His
top
bottom
CTR
scDb-hERG1
-β1-TRAIL
B
CTR
scDb-hERG1-β1
scDb-hERG1-β1-TRAIL
C
CTR
220
217
216
214
213
454
453
scDb-hERG1-β1-TRAIL
475
459
457
452
463
456
462
212

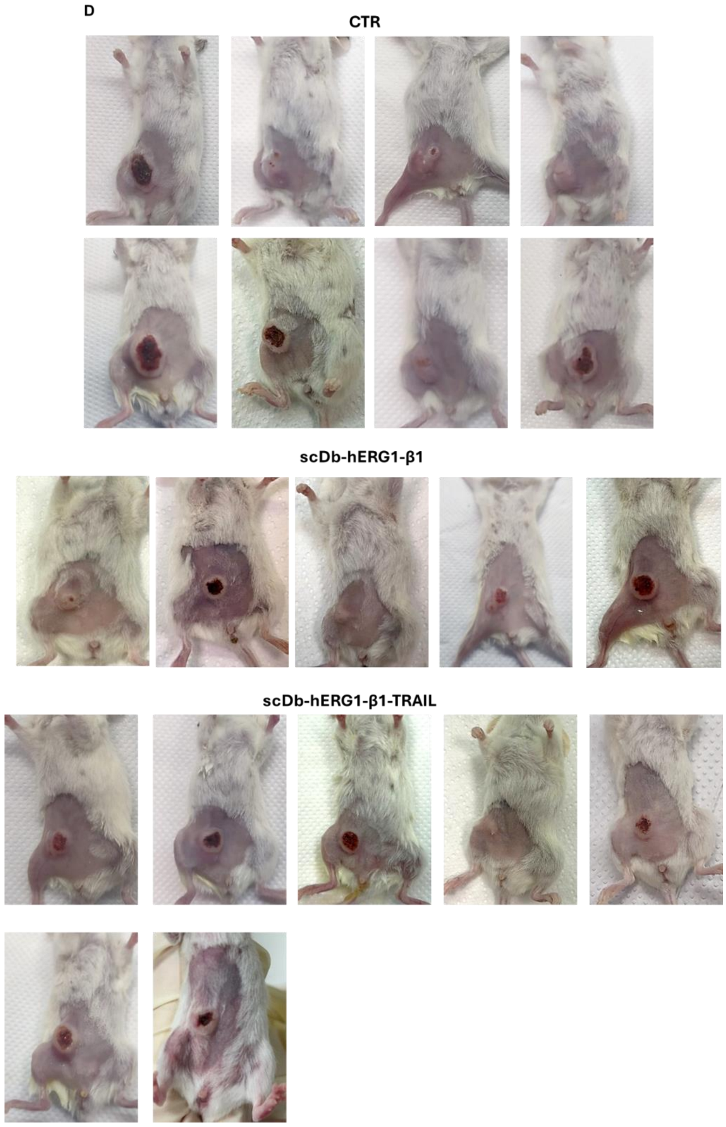

Supplementary Figure S6. In vivo data. A) Representative images and their respective enlargements of IHC staining with anti-6xHis antibodies of tumor masses obtained by subcutaneous injection of MDA-MB-231 cells treated with the scDb-hERG1-β1-TRAIL or with the vehicle (CTR). Scale bar, 200 µm. **B)** Representative high-resolution US images (left panels), PA images (right panels) of tumor masses derived from injection of MDA-MB-231 cells, treated with either the vehicle (CTR), scDb-hERG1-β1 or scDb-hERG1-β1-TRAIL, with the corresponding OxyHemo photoacoustic images; red areas indicate well oxygenated parts whereas blue and dark areas indicate the presence of hypoxia. Green ROI indicates necrotic area of the mass. **C)** Representative images of (Necropsy): Images of lungs from representative animals in which MDA-MB-231 cells were orthotopically injected in the breast. **D)** Representative pictures of masses at the end point before the explant. ROI: region of interest. PA: photoacoustic.
