# Supplementary material for: Antineoplastic Activity of a Novel Trispecific Single-Chain Antibody Targeting the hERG1/β1 Integrin Complex and TRAIL Receptors
Source: Mol Cancer Ther. 2025 Jun 18;24(10):1584–99. doi: 10.1158/1535-7163.MCT-24-0646 (PMC12485380; doi:10.1158/1535-7163.MCT-24-0646)
Supplement: Supplementary Table S1 — PRODUCTION OF scDb-TRAIL FUSION PROTEIN [file mct-24-0646_supplementary_table_s1_suppst1.pdf]

scDb FOR	**CAC CCA AGC TTG AGT CTG GAC CTG AAC TGG TGA**
Ts2/16 REV	**GACATGGTATAGCTACTGAAAG**
Ts2/16 FOR	**AGCCTCTGGATTCACTTTCAGTA**
scDb REV	**CC ACT TCC TCC TCC TCC ACT TCC TCC TCC TCC GGA TAC AGT TGG TGC AGC ATC**
TRAIL FOR	**AGT GGA GGA GGA GGA AGT GGC GGC GGC GGC TCT ATG GCC ATG ATG GAG GTC CAG**
TRAIL REV	**A GAA TGC GGC CGC GCC AAC TAA AAA GGC CCC GAA**

Supplementary Table S1. PRODUCTION OF scDb-TRAIL FUSION PROTEIN
