# Supplementary material for: Antineoplastic Activity of a Novel Trispecific Single-Chain Antibody Targeting the hERG1/β1 Integrin Complex and TRAIL Receptors
Source: Mol Cancer Ther. 2025 Jun 18;24(10):1584–99. doi: 10.1158/1535-7163.MCT-24-0646 (PMC12485380; doi:10.1158/1535-7163.MCT-24-0646)
Supplement: Supplementary Table S2 — Cycle steps [file mct-24-0646_supplementary_table_s2_suppst2.pdf]

scDb ISOLATION

- FIRST PART OF scDb isolation

scDb FOR

CAC CCA AGC TTG AGT CTG GAC CTG AAC TGG TGA

- sequence to facilitate cutting
- cutting site for **HindIII** A/ AGCTT

TTGCA /A

- to preserve frame
- **scDb sequence**

Ts2/16 REV

GACATGGTATAGCTACTGAAAG

- part that overlaps with Ts2/16 FOR
- **scDb sequence**

- SECOND PART OF scDb isolation

Ts2/16 FOR

AGCCTCTGGATTCACTTTCAGTA

- part that overlaps with Ts2/16 REV
- **scDb sequence**

scDb REV

CC ACT TCC TCC TCC TCC ACT TCC TCC TCC TCC GGA TAC AGT TGG TGC AGC ATC

- **Part of the linker that overlaps with TRAIL FOR in SOE-PCR**
- **Part of the Linker**

- **Part that overlaps with scDb**

TRAIL ISOLATION

We designed a couple of primers to isolate TRAIL from EGFP-TRAIL

TRAIL FOR

AGT GGA GGA GGA GGA AGT GGC GGC GGC GGC TCT **ATG GCC ATG ATG GAG GTC CAG**

- **Part of the linker that overlaps with scDb REV in SOE-PCR**
- **Part of the Linker**
- **Part that overlaps with TRAIL**

TRAIL REV

A GAA TGC GGC CGC GCC AAC TAA AAA GGC CCC GAA

- sequence to facilitate cutting
- cutting site for **NotI** GC/ GGCCGC

  CGCCGG/ CG

- **TRAIL sequence**

TRAIL: 281 ng/ul

scDb: 159 ng/ul

TRAIL ISOLATION with PCR (20ul)

	ul
**H_2O**	11,4
**5X PHUSION HF BUFFER**	4
**10 mM dNTPs**	0,4
**TRAIL FOR**	1
**TRAIL REV**	1

**TEMPLATE DNA**	2
**PHUSION DNA POLYMERASE**	0,2

Cycling Steps (1)

	°C	t
**Initial denaturation**	98	30s
**Denaturation**	98	10s
**Annealing**	60	30s
**Extension**	72	25s
**Final extension**	72	10min
	4	

25 cycles

The annealing temperature was calculated like:

Ta= 4°C (G+C)+ 2°C (A+T) à Ta= lower Tm- 4°C

The extension time was calculated like 30s/kb

scDb ISOLATION with PCR (20ul)

1) First part of scDb

	ul

**H_2O**	10,4
**5X PHUSION HF BUFFER**	4
**10 mM dNTPs**	0,4
**scDb FOR**	1
**Ts2/16 REV**	1
**TEMPLATE DNA**	3
**PHUSION DNA POLYMERASE**	0,2

Cycling Steps like (1)

2) Second part of scDb

	**ul**
**H_2O**	10,4
**5X PHUSION HF BUFFER**	4
**10 mM dNTPs**	0,4
**Ts2/16 FOR**	1
**scDb REV**	1
**TEMPLATE DNA**	3
**PHUSION DNA POLYMERASE**	0,2

Cycling Steps like (1)

FUSION CONSTRUCT

1) scDb2-TRAIL

	**ul**
**H_2O**	10,9

**5X PHUSION HF BUFFER**	4
**10 mM dNTPs**	0,4
**Ts2/16 FOR**	1
**TRAIL REV**	1
**TRAIL PCR product**	1,5
**scDb2 PCR product**	1
**PHUSION DNA POLYMERASE**	0,2

Cycle steps like (1)

2) scDb1- (scDb2-TRAIL)

	**ul**
**H_2O**	10,4
**5X PHUSION HF BUFFER**	4
**10 mM dNTPs**	0,4
**scDb FOR**	1
**TRAIL REV**	1
**scDb2-TRAIL PCR product**	2
**scDb1 PCR product**	1
**PHUSION DNA POLYMERASE**	0,2

Supplementary Table S2: Cycle steps: the extension time was 45” because of scDb2-TRAIL is longer than others amplified fragments. Others times like (1).
