# Supplementary material for: Antineoplastic Activity of a Novel Trispecific Single-Chain Antibody Targeting the hERG1/β1 Integrin Complex and TRAIL Receptors
Source: Mol Cancer Ther. 2025 Jun 18;24(10):1584–99. doi: 10.1158/1535-7163.MCT-24-0646 (PMC12485380; doi:10.1158/1535-7163.MCT-24-0646)
Supplement: Supplementary Table S3 — Peptides used for ELISA assays. [file mct-24-0646_supplementary_table_s3_suppst3.pdf]

PEPTIDE NAME	SEQUENCE
S5-P (hERG1)	EQPHMDSRIGWLHN (202404-0001 Primm s.r.l, Milano)
NK-12 (β1-integrin)	NKGEVFNELVGK (202404-0002 Primm s.r.l, Milano)
**PEPTIDE NAME**	**MAPPING RESIDUES**
DR4 TRAIL receptor	Pro34-Asn239 (TRL-HM2R1, Kactus)
DR5 TRAIL receptor	Ile56-Glu182 (DR5-HM201, Kactus)

Supplementary Table S3: Peptides used for ELISA assays.
