# Supplementary material for: Antineoplastic Activity of a Novel Trispecific Single-Chain Antibody Targeting the hERG1/β1 Integrin Complex and TRAIL Receptors
Source: Mol Cancer Ther. 2025 Jun 18;24(10):1584–99. doi: 10.1158/1535-7163.MCT-24-0646 (PMC12485380; doi:10.1158/1535-7163.MCT-24-0646)
Supplement: Supplementary Table S4 — IC50 values of the scDb-hERG1-β1, scDb-hERG1-β1-TRAIL and s-trimer-TRAIL on cell viability in different cell lines. [file mct-24-0646_supplementary_table_s4_suppst4.pdf]

CELL LINE	scDb-hERG1-β1 IC$_{50}$ (μM)	scDb-hERG1-β1-TRAIL IC$_{50}$ (μM)	s-trimer-TRAIL IC$_{50}$ (nM)
**HEK-293**	> 1.5	> 1.5	> 60
**HEK-hERG1**	0.22 ± 0.10	0.20 ± 0.16	> 60
**MCF-10A**	> 1.5	0.65 ± 0.14	6.03 ± 1.1
**MCF-7**	0.60 ± 0.12	0.35 ± 0.18	> 60
**MDA-MB-231**	0.35 ± 0.15	0.10 ± 0.11	5.40 ± 1.2
**U2932**	1.06 ± 0.13	0.75 ± 0.13	> 60

Supplementary Table S4: IC$_{50}$ values of the scDb-hERG1-β1, scDb-hERG1-β1-TRAIL and s-trimer-TRAIL on cell viability in different cell lines.
